# A Critical Review of Research on the Production and Properties of Chitosan Nanoparticles, Promising for Agrobiotechnology, Obtained Through Ionic Gelation with Sodium Tripolyphosphate

**DOI:** 10.3390/polym18131668

**Published:** 2026-07-06

**Authors:** Sergei L. Shmakov, Natalia N. Pozdnyakova, Oksana V. Tkachenko, Anna B. Shipovskaya

**Affiliations:** 1 Institute of Chemistry, Saratov National Research State University named after N.G. Chernyshevsky, 83 Astrakhanskaya St., Saratov 410012, Russia; shipovskayaab@yandex.ru; 2 Institute of Biochemistry and Physiology of Plants and Microorganisms, Saratov Scientific Centre of the Russian Academy of Sciences (IBPPM RAS), 13 Entuziastov Prosp., Saratov 410049, Russia; pozdnyakova_n@ibppm.ru (N.N.P.); oktkachenko@yandex.ru (O.V.T.)

**Keywords:** chitosan, tripolyphosphate, ionic gelation, nanoparticles, agrobiochemical applications

## Abstract

Nanoparticles of the aminopolysaccharide chitosan (ChNPs) are effective delivery platforms for biologically active substances for agrobiotechnological applications and hold great promise for solving precision problems in sustainable and efficient agriculture. This review presents an analysis of research publications during the past 20 years examining methods for producing ChNPs through ionotropic gelation using sodium tripolyphosphate for cross-linking macrochains, which are of practical interest for agriculture. Key aspects of the nanostructure formation process are analyzed, including the influence of the physicochemical characteristics of the aminopolysaccharide, the concentration and ratio of reagents, and ionic cross-linking conditions on the average size, size distribution (polydispersity), and zeta potential of nanoparticles. Particular attention is paid to several approaches proposed in the literature for determining optimal gelation conditions to obtain ChNPs with pre-specified size characteristics. Potential applications of nanostructured preparations based on these nanoparticles for agrobiochemical purposes are considered, including the encapsulation of antifungal, antiviral and antimicrobial agents, pesticides, NPK fertilizers, metal ions, plant extracts, essential oils, etc., to develop biodegradable stimulants for seed germination and plant growth, increased crop yields, and improved agricultural product quality. It is concluded that blocking the protonated amino groups of chitosan with tripolyphosphate anions is undesirable due to the reduced biological activity of the macromolecules and the nanostructured preparations obtained therefrom. An alternative approach for producing ChNPs with high biological activity with neither use of cross-linking agents nor encapsulation of agrochemicals is described.

## 1. Introduction

Currently, materials based on nanoparticles (NPs), whose size does not exceed 100 nm in at least one dimension, have been introduced and successfully used in such industries as electronics, power generation, construction, the chemical industry (nanocatalysts), and medicine (targeted drug delivery) [1,2,3,4]. Due to their valuable properties, they also have potential for application in electrical engineering [5], optics [6], biophotonics [7,8], sensorics [9,10,11], for the purification or decontamination of natural and industrial waters, environmental remediation, food packaging [12,13,14,15,16,17,18], and, finally, in agriculture for plant protection, increased crop yields, and improved soil quality [19,20,21,22,23]. For example, nanomaterials can be used to create delivery systems of agrochemicals and other biologically active ingredients (agromicroelements and their analogs, synthetic pesticides, selective strains of beneficial microorganisms for agrochemical applications, etc.) into plants or the topsoil [24,25,26,27]. Furthermore, nanostructured preparations can be used to functionalize bio(macro)molecules, which is a promising area at the intersection of nanotechnology and biotechnology [28]. However, the optimal option is when nanoparticles are based on biomacromolecules, thereby combining the properties of both carriers and active substance.

**Figure 1:**
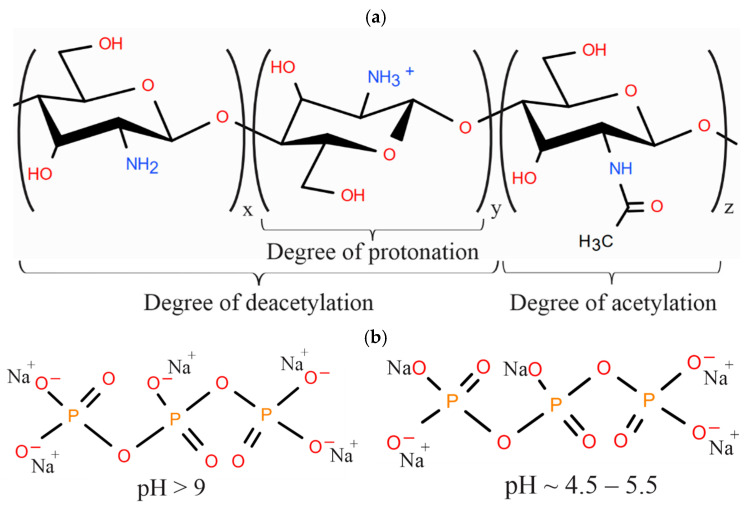
Molecular structures of (**a**) protonated chitosan and (**b**) tripolyphosphate polyanion at different pH values.

One of such biomacromolecules is the linear aminopolysaccharide chitosan (Ch), built from randomly distributed residues of D-glucosamine and N-acetyl-D-glucosamine linked by β-(1–4)-glycosidic bonds (Figure 1a) [29]. It is obtained by deacetylation of chitin, which has extensive sources in living nature (crustacean shells, insect exoskeletons, and some types of fungi). For several decades, Ch has been extensively studied due to constantly discovered new valuable properties and novel functional capabilities. Only low-molecular-weight (LMW) chitosan is soluble in water; otherwise, an acidic medium is required to protonate amino groups (i.e., to form polymer–acid salt complexes). In the overwhelming majority of cases, acetic acid (AcA) or an acetate buffer, or less commonly hydrochloric acid (HCl), is used to create such an environment. This process results in the formation of a water-soluble, protonated polymer form with positively charged amino groups ($-{\mathrm{NH}}_{3}^{+}$) on the macrochain, which determine the biological activity, primarily antimicrobial, of chitosan-containing materials [30,31,32,33].

Chitosan nanoparticles (ChNPs) were first obtained in 1994 by Ohya and co-workers [34] using an emulsion method involving cross-linking of Ch macromolecules with glutaraldehyde and mandatory ultrasonic treatment of the reaction system. In the original publication, they were called “chitosan-gel nanospheres”. Subsequently, various methods were developed for their preparation, namely: polyelectrolyte complex formation method [35], microemulsion method [36], emulsification solvent diffusion method [37], coacervation and reverse micellar method [38], ionic gelation [39,40], grafting of hydrophobic chains [41,42], etc. (see Table S2 in the Supplementary Information) Such a variety of methodological approaches makes it possible to obtain ChNPs with various morphologies, including spherical hollow and dense nanoparticles, agglomerate balls (clusters of several subunits) of spherical or anisodiametric shape, and nanofibers. Despite this, the overwhelming majority of publications use the ionotropic gelation method, which consists of cross-linking the protonated amino groups of Ch with polyanions of inorganic salts of tripolyphosphoric acid, due to, on the one hand, laboratory simplicity, and, on the other hand, high efficiency [43,44]. Nanoparticles form spontaneously, with no use of high temperatures, organic cosolvents, or ultrasonic treatment. The ChNP formation process is easily monitored by changes in the optical density (turbidity) of the system and requires neither recrystallization nor dialysis of the resulting nanoparticles. Therefore, the method is cost-effective.

However, to date, the research literature lacks reviews devoted to a detailed analysis of the key aspects of the process of nanostructure formation during ionic gelation, as well as the advantages and disadvantages of this method in the formation of chitosan nanoparticles of applied importance in agriculture. The influence of the physicochemical properties of the aminopolysaccharide, the quantitative ratio and concentration of reagents, as well as the conditions and parameters of ionic cross-linking on such characteristics of chitosan nanoparticles as their average size, size distribution and zeta potential has not been systematized. Existing review papers (see Table S1 in the Supplementary Information) focus primarily on the results of ongoing applied research on ChNPs with encapsulated ingredients (as important elements of agrobiotechnology), which are considered as a safe alternative for protecting agricultural crops from pests, diseases and stress factors, as well as plant growth stimulants that contribute to increasing crop yields [23,39,45,46,47,48,49,50,51,52,53,54]. Comparative studies of laboratory and industrial ChNPs with biologically active substances encapsulated are also being conducted, e.g., ChNPs with encapsulated garlic essential oil obtained by Candelo biotech SL (Albacete, Spain) [20,55]. In our view, it would be advisable to systematize the application of this method to reveal the maximum possible effect it can provide, primarily the minimum size of ChNPs, low polydispersity index (PDI) and optimal zeta potential, which provide an increased surface area and size effects [30], and also to provide an overview of the possible applications of this type of chitosan-containing nanoparticles. These size and electrochemical characteristics are extremely important for agrochemical applications, since due to their small size, ChNPs acquire additional valuable properties. Thus, this review analyzes and systematizes the latest advances in the production, properties, and application of chitosan nanoparticles obtained by ionotropic cross-linking of macrochains, which hold promise for agrobiotechnology.

The ionotropic gelation method is based on electrostatic interactions between the positively charged protonated amino groups of chitosan and the negatively charged phosphate groups of sodium tripolyphosphate (TPP), the most popular ionotropic cross-linking agent due to its non-toxicity to the human body and low cost (Figure 1b). Although this method can form a gel across the entire reaction vessel, with appropriately selected concentrations and ratios of reagents, as well as other conditions, a gel-like structure with a 3D cross-linked network within one or more macromolecules could be formed as individual nanoparticles (such systems are sometimes referred to as nanogels in the literature).

A standard technique for obtaining ChNPs has been developed. First, Ch is dissolved in an acid (usually AcA) and, as a rule, the pH of the solution is increased (to reduce swelling of the polymer coils) with an alkali solution [56,57,58]. In order to reduce the toxicity of the alkaline neutralizing agent, an organic base (triethanolamine) can be used, but only a few studies are devoted to this [59,60]. A TPP solution of a given concentration (*C*_TPP_) is added dropwise to a solution of Ch in AcA with given concentrations (*C*_Ch_, *C*_AcA_) (To maintain consistency in metrological characteristics, the concentrations of Ch, AcA, and TPP are given in g/dL throughout this paper, regardless of the source of information), previously brought to the required pH value. We note in passing that the authors provide either specific volumes of the Ch and TPP solutions (*V*_Ch_, *V*_TPP_), or their ratio *V*_Ch_:*V*_TPP_ (the latter is most correct). The solutions of the aminopolysaccharide and the ionotropic agent, as a rule, should have similar pH values, which for an aqueous TPP solution is achieved by acidification with HCl. A controlled rate of TPP addition to the Ch solution is also required to ensure the gradual formation of nanosized particles without their aggregation up to micrometer sizes [61]. Ultrasonication to prevent aggregation of nanostructures, centrifugation and other processing methods to improve the quality of the resulting nanoparticles are then possible. After ion titration, the morphological form of ChNPs is assessed, their zeta potential, size with polydispersity or size distribution are determined, most often by dynamic light scattering (DLS), atomic force microscopy (AFM), scanning electron microscopy (SEM) or transmission microscopy (TEM), less often by X-ray powder diffraction (XRPD) with calculations using the Debye–Scherrer equation, laser diffraction (LD), and differential centrifugal sedimentation (DCS). ChNPs obtained by ionotropic cross-linking are primarily spherical or nearly spherical. Their sizes, estimated by various methods, typically vary, owing not only to some details of a particular method but also to some properties of the particles. For example, the difference between the hydrodynamic diameter of optimized ChNPs in an aqueous medium (138 nm, DLS) and their actual diameter in an air-dry state (30–50 nm, TEM) is due to particle swelling in water [58].

Due to differences in the principles underlying these methods, the sizes of the same particles estimated by them always differ. Such methods as Nanoparticle Tracking Analysis (NTA) and Multi-Angle Light Scattering (MALS) combined with Field-Flow Fractionation (FFF) offer the highest accuracy but are rarely used due to the complexity of the methodology and the lack of necessary equipment. In addition, the use of ultra-precise techniques is only justified when particle sizes remain constant despite variations in experimental conditions, such as the rate of dropping of one solution into another. Rational use of the obtained ultra-precise results is also necessary, e.g., for experimental design, which is not always the case in research works.

Due to the similarity of the methods, the results of many studies are systematized in a table (Table A1). ChNP characteristics were generally determined under standard room-temperature conditions. The last column lists chemicals used (or potentially promising for use) in agricultural technologies, encapsulated in nanoparticles. The papers are arranged mainly in order of increasing average sizes (*d*, nm) of ChNPs, although it is hardly possible to maintain this order precisely, since Ch samples with different physicochemical characteristics were used, namely, molecular weight (MW, kDa), degree of deacetylation (DD, %) or acetylation (DA = 100 − DD, %), reflecting the number of glucosamine or N-acetylglucosamine units to the total number of monomer ones. The methods for assessing the size parameters of the nanoparticles also varied; sometimes, not a single size, but a whole range is given. Acetic acid was mainly used as a solvent. In some cases, ascorbic or citric acids, or acetate buffer were taken. Many researchers often borrow the procedure for obtaining ChNPs from other studies [62,63,64,65], since the experiments they conduct are of a purely applied nature.

A number of studies examine ionotropic gelation in great detail, with particular attention paid to analyzing the influence of various factors on some properties (average size, size distribution, polydispersity, and zeta potential) of the resulting nanoparticles. Let us examine the key characteristics of ChNPs that are subject to regulation during their production by ionic cross-linking, as discussed in these papers.

## 2. Average Size of Chitosan Nanoparticles

The average size of the resulting ChNPs is significantly affected, first of all, by the average statistical conformation of the macromolecular coils of protonated Ch in solution, the average distance between them, and the amount of TPP added (the stoichiometric ratio between the amino groups of Ch and the phosphate groups of the TPP anions—NH_2_/PO_4_) (This refers to the gross concentrations of the reagents, without considering the degree of protonation/dissociation). The macromolecular conformation is known to be mainly determined by the molecular weight of the polymer. The latter, in turn, correlates with the hydrodynamic diameter of the macrocoil in solution and the size of the resulting nanoparticles, namely, the smaller the macrocoil, the smaller the ChNPs. Therefore, the use of an LMW polymer or oligomers allows obtaining stable suspensions of ChNPs below ~100 nm in size [66,67,68,69,70]. At the same time, Ch samples with a higher MW may form micron-sized aggregates and less stable suspensions [68,71,72,73]. On the other hand, before cross-linking, the coils should not only correspond to the optimal degree of swelling, but also contain a sufficient number of ${\mathrm{NH}}_{3}^{+}$—groups to react with TPP anions (Figure 1), which is achieved by introducing specially selected amounts of an alkaline agent. An excessive coil size, due to polyelectrolyte swelling, reduces the average distance between the coils and, thereby, increases the probability of cross-linking of adjacent macrochains. These factors depend not only on the MW and DD of the aminopolysaccharide, but also on some external conditions, such as the concentration of Ch and TPP solutions, the concentration of the Ch acid solvent, the Ch:TPP ratio, temperature, pH and ionic strength of the reaction medium, and the stirring speed. The latter should be 600–800 rpm to prevent particle aggregation [57,61]; higher stirring speeds provoke the formation of large aggregates, ~1 μm [58]. Furthermore, the stirring rate during ionic gelation has a significant impact on the reaction efficiency [61]. Therefore, by regulating this parameter, it is possible to significantly increase the yield of nanoparticles with a pregiven size range. The aggregation and sedimentation stability of ChNPs over time are also studied. Thus, the current research is aimed at revealing optimal conditions to facilitate the formation of nanoparticles of minimal size and, consequently, maximum activity. The following subsections provide a more detailed analysis of the influence of the abovementioned factors on the size characteristics of ChNPs.

### 2.1. Effect of the Concentration of Chitosan Solutions, Organic Acid, and Ionotropic Cross-Linking Reagent

The size of chitosan nanoparticles obtained by ionotropic gelation using a sodium tripolyphosphate solution depends on both the concentration of the aminopolysaccharide and the aqueous-acidic medium used for its protonation, as well as the ionotropic agent concentration (Table A1).

As a rule, the size of ChNPs changes symbatically with the Ch concentration in the solution which the TPP solution is added dropwise into. A decrease in the concentration of Ch leads to a decrease in the size of the resulting particles [56,65,68,74,75], although for the data in Table A1, this trend is obscured by differences in other characteristics and conditions. For example, Divya et al. [75] studied the dependence of the average ChNP sizes on a number of factors, including *C*_Ch_. The MW of the Ch sample used is not specified, but an LMW sample was probably used. At a concentration within 0.1–0.2 g/dL, the average size of ChNPs was ~20 nm, and within *C*_Ch_ = 0.3–0.5 g/dL it was 120–165 nm. In addition, the size of the nanoparticles increased with the ionic cross-linking reaction time, being minimal 60 min after its start. Jonassen et al. [74], using LMW chitosan hydrochloride (PROTASAN UP Cl 213) as an example, also established that larger particles were formed at higher chitosan concentrations: with an increase in *C*_Ch_ from 0.05 up to 0.10 g/dL, the *d* values increased more than 2-fold.

**Figure 2:**
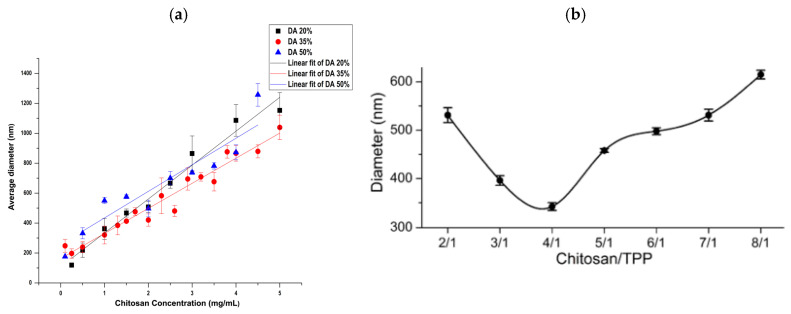
Effect of the physicochemical parameters of chitosan and the components of the ionotropic gelation reaction on the size characteristics of ChNPs. (**a**) Effect of chitosan concentration on the average hydrodynamic diameter of particles obtained at the degree of acetylation (DA) of 20% (■), 35% (●) and 50% (▲) with the –NH_2_/PO_4_ ratio of 1.5 for DA of 20% and 50%, and with the –NH_2_/PO_4_ ratio of 1.0 for DA of 35%. The *R*^2^ values were 0.97, 0.93 and 0.82 for DA of 20%, 35% and 50%, respectively [56]. (**b**)—Dependence of the average diameter of ChNPs on the chitosan/TPP ratio [40]. Licensed under Creative Commons CC BY.

A similar pattern of change in *d* with increasing *C*_Ch_ was observed for high-molecular-weight (HMW) Ch samples. Ing et al. [68] used two chitosan samples with a MW of 70 kDa, DD of 75–85% and 310 kDa, 85%. The average size of ChNPs increased with both concentration (0.1–0.3 g/dL) and molecular weight of the polymer. At the same time, the nanoparticles based on LMW Ch showed a relatively narrow size distribution and low PDI values (0.4–0.6) over the entire range of *C*_Ch_, while those based on HMW Ch—at *C*_Ch_ = 0.1 g/dL only; while at *C*_Ch_ = 0.2 and 0.3 g/dL the PDI was as broad as 0.8–1.0. Sreekumar et al. [56], using samples with a MW within 125–450 kDa and a DD within ~40–98.5%, but with *C*_Ch_ = 0.05–0.5 g/dL close to the previous work, confirmed a direct correlation between the Ch concentration and the average hydrodynamic diameter of the nanoparticles (Figure 2a). The average size of ChNPs varied in the range of ~100–1200 nm with low polydispersity (0.1–0.4). Based on the concentration dependence of the intrinsic viscosity [η] and the macrocoil overlap concentration, it was established that particles with the smallest hydrodynamic diameter (~100 nm) were formed in solutions at *C*_Ch_ corresponding to the condition *C*_Ch_[η] < ~4. Apparently, at low Ch concentrations, TPP anions preferentially bind the amino groups of the same macromolecule, while with increasing concentrations and the average proximity of the coils, the probability of ionic cross-linking of several adjacent macromolecules increases. An increase in the initial size of ChNPs with increasing initial Ch solution concentration (medical-grade Ch powder, DD 90%, *C*_Ch_ = 0.2–0.6 g/dL) was also noted by Handani et al. [76].

In the case of highly deacetylated Ch, an increase in *d* and PDI with increasing *C*_Ch_ was observed at lower polymer concentrations, e.g., according to Dong et al. [65], an increase in the average ChNP size from ~150 up to ~380 nm and an increase in PDI from 0.15 up to 0.46 with increasing Ch solution concentration (MW 200–500 kDa, DD 91.8%) was already observed in the range *C*_Ch_ = 0.025–0.1 g/dL. Despite the broadening of the particle size distribution for larger ChNPs, the PDI generally corresponds to the characteristics of monodisperse nanoparticles. The authors attribute this to the ease of penetration of TPP anions into chitosan macrocoils. At low concentrations, macromolecules adopt extended conformations due to repulsion between their positively charged protonated amino groups remaining after pH adjustment with an alkaline agent. Within one macrocoil, there is sufficient space for rapid movement of TPP anions. Therefore, subsequent cross-linking ensures the formation of smaller ChNPs with a more uniform size distribution. As the Ch concentration and, consequently, the number of macromolecules increase, the latter move closer together and begin to adhere to each other and aggregate. Unfortunately, the authors of the studies discussed in this subsection established no lower concentration limit (i.e., the limit to which it is advisable to reduce the Ch concentration). It is most likely that the lowest *C*_Ch_ should also be determined taking into account the polymer’s MW.

To reveal the optimal *C*_Ch_ for obtaining ChNPs with pregiven size parameters, Francisco Goycoolea’s research team proposed using the dimensionless space occupancy degree *C*_Ch_[η] instead of the mass-volume Ch concentration, which takes into account the degree of contact among macromolecular coils before cross-linking [56,77]. Involving the intrinsic viscosity [η] allows simultaneously considering the concentration, average molecular weight, and degree of deacetylation of Ch (see also Section 2.2). Unfortunately, this approach was applied only to a small number of Ch samples with a narrow variation in their [η] values. In addition, the authors did not plot a master curve: the experimental lines on the dependence of the average nanoparticle diameter on *C*_Ch_[η], obtained for three Ch samples with the same DA (12%) and different average viscosity MW (2.5, 31.2, and 139.2 kDa), diverge when *C*_Ch_[η] > 2.5 [77]; the experimental points of the curve *d* = *f*{log(*C*_Ch_[η])}, also obtained for three Ch samples with the same DA (36%) and different Mw’s (125, 280, and 450 kDa), form a wide corridor when log*C*_Ch_[η] > 10 [56]. However, this approach appears to be very promising and can be recommended for use by future authors in their work.

The size of chitosan nanoparticles can also be regulated by changing the concentration of the acid solvent of the polymer, as well as the ionotropic agent concentration. For example, in a Ch solution (LMW sample, DD 91.5%) with *C*_Ch_ = 0.05 g/dL and *C*_AcA_ = 0.01 g/dL, ChNPs with *d* = 196 nm and PDI = 0.2 were formed [58]. Increasing *C*_AcA_ to 0.02–0.05 g/dL in the Ch solution of the same concentration decreased *d* to 138 nm, and PDI to 0.03–0.08 (Table A1). At a high molar AcA excess (*C*_AcA_ = 0.08 g/dL), the average size of the formed ChNPs remained unchanged (138 nm), but their PDI increased, albeit only to 0.12, i.e., within the limits of monodispersity. Thus, increasing *C*_AcA_ within the range of 0.01–0.08 g/dL led not only to an increase in the number of small particles (weakening of the polyelectrolyte effect due to the influence of ionic strength and compaction of macrocoils), but also to an increase in the proportion of large particles (weakening of intermolecular repulsion, aggregation of adjacent macromolecules), which deteriorated the monodispersity of the system, albeit slightly. A decrease in the size and polydispersity index of ChNPs with an increase in the concentration of AcA used in the preparation of the aqueous Ch solution (LMW sample, DD > 95%) was also observed by Abdel-Hafez et al. [57]. Since the authors of both studies used LMW samples, this behavior may be due to increased polymer solubility because of the effective destruction of inter- and intramolecular bonds in the supramolecular Ch structure.

An increase in the TPP concentration, all other things being equal, increases the size of ChNPs [58]. In the study discussed, the optimal value for obtaining nanoscale structures was *C*_TPP_ = 0.05 g/dL; when *C*_TPP_ > 0.1 g/dL, microparticles were formed. Therefore, the TPP concentration should be optimized relative to the Ch concentration. It has also been shown that the critical TPP concentration depends on the molecular weight and degree of deacetylation of Ch [77,78]. For efficient cross-linking of the macrocoils of HMW chitosan, a higher TPP concentration is required, but this may result in the formation of larger particles compared to LMW chitosan.

### 2.2. Effect of the Degree of Deacetylation of Chitosan

The influence of the degree of deacetylation on the size of chitosan nanoparticles was already partially discussed in Section 2.1. We further note the following. Sreekumar et al. [56] found that with a decrease in the DD (i.e., a DA increase) of chitosan, the slope of the linear approximations of the dependence of the average diameter of ChNPs on the polymer concentration decreases (Figure 2a). This could be explained by a decrease in the number of amino groups per unit volume, similar to a decrease in the polymer concentration in solution (unfortunately, the average MWs of chitosan at these DDs differ). Their studies also show that, due to the different numbers of amino groups available for cross-linking in Ch macrochains with different DDs, the TPP concentration should be optimized for every polymer sample to achieve the minimum particle size.

Huang et al. [79] plotted a series of average diameter–TPP:glucosamine unit ratio dependencies for Ch samples with MWs within 57–76 kDa and DDs within 63–95%. At high DDs (82 and 95%), the average hydrodynamic particle diameter gradually decreased to a certain TPP:glucosamine unit ratio (~0.22:1 and ~0.28:1, respectively), and then sharply increased, indicating ChNP aggregation or cross-linking of adjacent coils. At relatively low DDs (63 and 72%), aggregation was not observed across the entire range of TPP concentrations studied; only a gradual decrease in the average ChNP size (additional cross-linking within the existing particles) occurred. Thus, the presence of acetamide units weakens ionotropic gelation. This is initially more pronounced during cross-linking of adjacent coils (the abscissa of the growth onset shifts to the right), but then it also begins to manifest itself during cross-linking within a single coil: less cross-linked ChNPs with a DD of 63% were somewhat larger than more cross-linked ChNPs with a DD of 72%. Therefore, the optimal (for the Ch samples studied in this work) DD of chitosan can be considered to be in the range of 72–82%. However, Pham et al. [67] were able to obtain ChNPs with an average diameter of 25–30 nm (TEM) even with a DD of 90% (Table A1). An increase in the diameter of nanoparticles with a decrease in the DD of chitosan was also noted in Ref. [80].

Conclusions similar to those presented above were made by Kleine-Brueggeney et al. [77], where Ch samples with similar MWs of 162–182 kDa and a wide range of DA variation of ~0–47% were used to obtain nanoparticles. Two ranges of ChNPs size were distinguished depending on the DA of the aminopolysaccharide, namely, large nanoparticles with *d* = 300–400 nm for samples with DA ~ 0–22% and small ones with *d* = 90–140 nm when DA ~ 35–47%. Moreover, the influence of the ratio of N-acetylglucosamine and N-glucosamine units to the total number of monomer units in the macrochain on the properties of ChNPs was considered indirectly, through the influence of DA on the conformational parameters of Ch macromolecules before cross-linking. The authors determined the intrinsic viscosity [η] of chitosan solutions and calculated the degree of volume occupancy *C*_Ch_[η] by macromolecular coils, i.e., they essentially found the reduced concentration of macrocoils per unit volume. Ch samples with DA < 22% formed nanoparticles only when *C*_Ch_[η] did not exceed the threshold value of 1.3, corresponding to the coil overlap onset. But higher *C*_Ch_[η] values were required to obtain ChNPs using samples with DA > 34%. This is clearly illustrated in the graphical abstract of this work [77]. The authors believe that with increasing DA, Ch macromolecules become more flexible and their polyelectrolyte charge decreases, leading to coil compaction and the need to use Ch solutions of higher concentrations to obtain ChNPs. As a criticism, we note that achieving the minimum ChNP size can only be a goal under otherwise equal conditions, since increasing DA (decreasing DD), even while providing small nanoparticle sizes, reduces the number of active amino groups in the macromolecule and thereby reduces the functional value of the resulting nanostructures.

From the above, it can be concluded that the degree of deacetylation/acetylation determines the positive charge density of chitosan macrochains, namely, the higher the DD (lower the DA), the more free amino groups are available for protonation and electrostatic interaction with TPP [56,79,80]. This regularity has a direct impact on the process of ionotropic gelation, which, in turn, determines the size of the resulting nanoparticles. The effect of the ratio of N-glucosamine and N-acetylglucosamine units in the macrochain on the size of ChNPs is most pronounced for HMW Ch, while for LMW Ch the particle size may remain relatively unchanged with varying DD/DA [81]. While the DD/DA ratio is certainly significant, it is not the aminopolysaccharide’s only parameter affecting the size of ChNPs. The ratio of reactants in the ionotropic gelation reaction also plays a critical role in optimizing the nanostructuring of chitosan macrochains.

### 2.3. Effect of the [–NH_2_]/[PO_4_] Ratio

Since ChNPs are synthesized in a significantly non-equilibrium mode, many authors describe their specific method by providing not the final concentrations of reactants in solution, but the concentrations and volumes of the initial solutions, and sometimes only the ratio of these volumes. However, it would be more accurate to represent the reactant concentration as the [–NH_2_]/[PO_4_] gross ratio, since it is quite difficult to account for the degree of protonation of the amino groups of Ch and TPP dissociation.

Fan et al. [58] used 0.05 g/dL solutions of LMW Ch (DD 91.5%) in 0.02 g/dL AcA (with pH pre-adjusted to 4.7–4.8 with 20% NaOH) and assessed the effect of the volume of 0.05 g/dL TPP added dropwise to 10 mL of the chitosan solution on the size of ChNPs. As the TPP solution volume increased from 2.5 up to 3.5 mL (Ch:TPP mass ratio from 4.0:1 to 2.9:1), the particle size initially decreased smoothly in the range of 175–130 nm (binding of individual macromolecules and formation of additional bonds within the formed particles), and then sharply increased up to 240 nm (binding between different macrocoils and particle aggregation began). Abdel-Hafez et al. [57] found that with a decrease in the CS:TPP ratio, the average diameter of NPs passed through a gentle minimum, and PDI decreased. An LMW Ch sample with a high DD > 95%, *C*_Ch_ = *C*_AcA_ = 0.1 g/dL, *C*_TPP_ ~ 0.06 g/dL was also selected for the study. Using a volume Ch:TPP ratio = 2.5:1–4:1 led to the formation of nanoparticles with the minimum sizes (*d* ~ 60–70 nm) and the narrowest polydispersity (PDI ~ 0.15–0.25). A similar effect of the [–NH_2_]/[PO_4_] ratio on the size of ChNPs was found for HMW Ch (MW 350 kDa, CD > 75%) [40]. In this case, solutions of a polymer concentration comparatively high for HMW Ch, *C*_Ch_ = 0.2 g/dL, were studied. Despite the fact that the resulting dependence was plotted in the reverse order (Figure 2b, Ch:TPP rather than TPP:Ch on the abscissa axis) compared to a similar dependence from Ref. [58], it demonstrates a similar tendency for the ChNPs diameter to pass through a minimum value. The best reagent ratio for obtaining ChNPs with minimal *d* and PDI was Ch:TPP = 4:1 (in this specific case). At Ch:TPP = 3:1, the particle size was slightly higher, but the PDI was also narrow unimodal. Thus, the volume ratios of Ch:TPP = 3:1–5:1 can be considered optimal for this specific case, which is consistent with other results [57,70,82]. At higher Ch:TPP values, aggregation, a decrease in zeta potential, and, accordingly, kinetic instability of particles up to their sedimentation were observed; at lower values, unstable particle formation occurred due to an excess of anionic charges in the system compared to cationic ones [56].

To obtain smallest-sized ChNPs, researchers used various optimization methods, including approximation by algebraic equations. For example, a central composite design was proposed to optimize the conditions for ChNP production [71]. The results of 13 experiments for a Ch sample with an MW of 1129 kDa and a DD ≥ 75% were approximated by the equation:

$$\sqrt{size}=9.95-3.26A+1.51B-2.99AB+3.80A^{2}+2.11B^{2},$$

where the independent variables *A* and *B* are the concentration of Ch (0.05–0.15 g/dL) and TPP (0.05–0.10 g/dL), respectively. As *C*_TPP_ increased, the size of ChNPs passed through a minimum, as in Refs [40,57,58]. The authors offer the following explanation: partial neutralization of the charge of chitosan amino groups by the phosphate groups of the ionotropic agent reduces the repulsion of positive charges in the macrocation and, accordingly, the macrocoil size. With further addition of TPP, complete neutralization of the positive charges occurs (similar to the polyelectrolyte effect suppression), which removes mutual repulsion and leads to ChNP aggregation. A batch of ChNPs optimized using this equation had a particle size of ~100 nm at a Ch concentration of 0.1% and a TPP concentration of 0.08%.

A number of studies have used not only pH regulation but also an increase in the ionic strength of the medium by introducing a strong neutral salt to obtain compact ChNPs. For example, Jonassen et al. [74] simultaneously investigated three factors influencing nanoparticle properties, namely: chitosan concentration (0.05 and 0.10%), Ch:TPP volume ratio (95:5, 90:10, 85:15, and 80:20), and the ionic strength of the solvent (water, 0.05, and 0.15 M NaCl)—a total of 24 combinations. The weight-average (*M*_w_) and number-average MW (*M*_n_) of chitosan were 307 and 115 kDa (PROTASAN), respectively, with a DD of 83%. NaCl introduction into the initial Ch solution significantly compressed the macrocoils (due to an increase in the ionic strength of the solution and enhanced Debye screening of the charged amino groups of chitosan) and, accordingly, reduced the size of the resulting ChNPs, the greatest effect being observed at a moderate salt concentration. At a low Ch concentration (0.05%) and high Ch:TPP ratios (95:5 and 90:10), the nanoparticles were stable over time, but at low Ch:TPP ratios (85:15 and 80:20), sedimentation occurred after a month of storage. At a high Ch concentration (0.1%), nanoparticles could be obtained without subsequent sedimentation at the highest Ch:TPP ratios only (95:5 and 90:10). However, after a month of storage, the average hydrodynamic radius of ChNPs obtained at a Ch:TPP ratio of 90:10 decreased significantly. Additional binding of free protonated amino groups by TPP anions apparently occurred over time. At low CS:TPP ratios, the particles were expected to be unstable over time, and their zeta potential decreased. It should also be noted that the production of compact NPs with *d* ~ 150 nm and a narrow size distribution by introducing low NaCl concentrations (0.05 g/dL) into the initial Ch solution (MW 100 kDa, DD 90%) was reported in Ref. [82].

### 2.4. Effect of Temperature

Temperature influences ionotropic gelation through a complex of interrelated factors. First, an increase in temperature accelerates the diffusion of cross-linking reagent ions and promotes faster nanoparticle formation [78]. Second, temperature affects the flexibility of polymer chains and their ability to form intra- and intermolecular bonds with the phosphate groups of the cross-linker [56]. Finally, a change in temperature may alter the solubility of Ch in AcA and TPP in water [58]. Overall, our analysis of the available literature showed that temperature had a variable effect on ChNP sizes, depending on other conditions and parameters of ionotropic cross-linking, as well as the physicochemical characteristics of the aminopolysaccharide, the concentration and ratio of the reactants, and the acidity of the medium.

According to Fan et al. [58], the size of nanoparticles (measured at 25 °C) showed a clear tendency to increase as the temperature of the chitosan solution decreased from 70 down to 10 °C during the cross-linking of macrochains. As noted in Section 2.1, dilute LMW Ch solutions with *C*_Ch_ = 0.05 g/dL were used; in this case, *C*_AcA_ = 0.02 g/dL. The vessel with a Ch solution was first heated and then placed onto a magnetic stirrer in a freezer at 2–4 °C. A TPP solution pre-cooled to 2–4 °C was added, and the cross-linking reaction was carried out for 10 min. In the range of 70–60 °C, the size of ChNPs was minimal (136 ± 1 nm), while in the range of 55–10 °C, it increased up to 200 nm as the reaction system cooled. Within 70–25 °C, the PDI values of the resulting particles were below 0.05, while at 10 °C PDI = 0.11. Since the Ch solution viscosity decreases with heating, the small values of *d* and PDI may be due to a decrease in the radius of gyration of the macromolecules and the amount of hydrogen-bonded water with increasing temperature. This, in turn, leads to increased flexibility of the polymer chain and a decrease in the specific volume of the chitosan molecule. Both effects, as well as the possible decrease in the thermodynamic quality of the dissolving medium under these conditions, apparently contribute to some compression of macromolecular coils, the formation of a compact structure during cross-linking, and, consequently, the formation of small-sized ChNPs. At the same time, similar experiments with the addition of a cold TPP solution to a hot Ch solution, carried out at 10–15 and 16–25 °C, demonstrated that low temperatures (2–4 °C) of the ionotropic gelation process improved the monodispersity of ChNPs (Table A1). The authors explain these characteristics of the resulting ChNPs by the high cooling rate of the reaction system at low temperatures, as well as the strengthening of hydrogen bonding between the polar groups of the polymer and water molecules, which prevent the cross-linking of adjacent macrochains.

However, Abdel-Hafez et al. [57] established an inverse temperature dependence of *d*. But the ionotropic gelation conditions were also different. The reaction temperature (in the range 4–50 °C) was set using a thermostatically controlled water bath and remained unchanged during cross-linking. The physicochemical characteristics of Ch and the [–NH_2_]/[PO_4_] ratio were similar to those in the previous paper, but solutions with *C*_Ch_ = 0.1 g/dL were used. In the temperature range 4–30 °C, the average size of the resulting particles was ~80 nm, being virtually independent of the gelation temperature, while PDI decreased from ~0.25 down to ~0.15 as the temperature increased. This could be explained by a decrease in the internal energy of the particles required for collisions and subsequent aggregation (it should be noted that Handani et al. [76] also found that temperatures within 15–30 °C during ionotropic gelation did not significantly affect the initial size of the resulting ChNPs). When cross-linking was carried out at 50 °C, an increase in the diameter of ChNPs up to 300 nm was observed with a simultaneous decrease in PDI down to ~0.05. This is likely due to a TPP excess and/or a high Ch concentration, which contribute to an increased probability of intercoil binding. In addition, the authors plotted 3D surfaces that clearly demonstrate the effect of the Ch:TPP mass ratio and the pH of the Ch solution at three temperatures on the diameter of ChNPs. At a low temperature (4 °C) within the studied range, there is a minimum at a Ch:TPP ratio of 3.8:1 and pH 4.4. A similar relationship between *d* and PDI, caused by the influence of pH, was obtained in Ref. [83].

The size characteristics of Ch nanoparticles are also affected by the storage temperature of the nanosuspension. For example, Morris et al. [78] studied the effect of long-term storage at different temperature conditions (4, 25, and 40 °C) on the size of ChNPs and their size distribution using the DCS method. Ch samples from Pronova Biomedical (Oslo, Norway) with a lower DD (~80%) than in Refs [57,58] were used to obtain nanoparticles. It was found that during 12 months of storage at 4 and 25 °C, the hydrodynamic diameter of ChNPs remained unchanged compared to the *d* of freshly prepared nanoparticles and was 110 ± 40 nm. The particle size distribution also showed minimal variability. At the same time, significant degradation of the particles was recorded at a storage temperature of 40 °C. After just one month of storage, a decrease in the nanoparticle size and a narrowing of their distribution were observed, and after six months, no ChNPs were detected. The main cause of instability of this type of ChNPs at elevated storage temperatures may be destruction of the polymer network due to increased thermal motion and weakening of the electrostatic interactions of Ch polycations with TPP anions. However, other mechanisms that could contribute to instability cannot be ruled out, such as changes in the degree of ionization of functional groups, hydrolysis of phosphate bonds, or structural reorganization of polymer coils.

### 2.5. pH Dependence

The pH of the medium is a critical factor determining the size and other physicochemical characteristics of ChNPs obtained by ionotropic gelation and affecting their stability. The mechanism of this effect is associated with changes in the degree of ionization of the functional groups of both Ch and TPP, which directly influences the cross-linking process and the formation of nanoscale structures. With pH below 6.0, the amino groups in the Ch macromolecule are protonated, forming a polycation ($\sim {-\mathrm{NH}}_{3}^{+}$) highly soluble in water and capable of ionic cross-linking with the negatively charged phosphate groups of the ionotropic agent (see Figure 1). As the pH increases above the p*K*_a_ of the amino groups, the Ch solubility decreases, which may lead to precipitation and coagulation of the polymer substance instead of the formation of homogeneous nanoparticles. Therefore, high-concentrated acid (above the minimum required) is typically used to dissolve Ch. However, in a molar excess of acid, the polymer coils swell due to the polyelectrolyte effect, which hinders the ionic binding of protonated amino groups within a single macromolecule. Due to competition, the probability of cross-linking of the amino groups of adjacent chains increases, leading to the formation of larger ChNPs. To reduce coil swelling, the pH of the initial Ch solution is increased by carefully adding an alkaline reagent, preventing the polymer from precipitating into a coarse precipitate. It can be assumed that, as with the Ch:TPP ratio, there is an optimal pH value. E.g., the examples given in Table A1 use pH in the range of 4.5–5.5.

**Figure 3:**
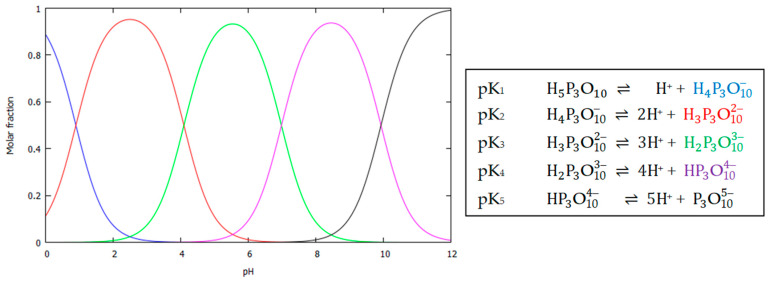
pH distribution of the charged forms of TPP (calculation was performed by the authors of this paper directly from the equilibrium equations, mathematical package MAXIMA).

We should also consider TPP protonation at different pH levels, since this process significantly affects its chemical properties and, consequently, its ability to electrostatically interact with counterions. The molecular structure of the sodium salt of tripolyphosphoric acid includes three phosphate groups sequentially linked via oxygen atoms. TPP protonation in an aqueous medium is a complex multi-stage acid–base process involving sequential addition of protons to the oxygen atoms of the phosphate groups (Figure 3). Protonation occurs predominantly at the terminal phosphate groups, which is associated with their increased basicity compared to the internal phosphate ones [84]. The first two protonation stages proceed in a strongly acidic medium, the third in a slightly acidic medium, and the last two in neutral and alkaline media, respectively. The dissociation constants of tripolyphosphoric acid were determined by Edwards et al. [85]: pK_2_ = 0.89 ± 0.57, pK_3_ = 4.09 ± 0.08, pK_4_ = 6.98 ± 0.02, pK_5_ = 9.93 ± 0.04. Since the pH of an aqueous-acidic solution of Ch is usually between p*K*_3_ and p*K*_4_, it can be concluded that the TPP anion (${{H_{2}P}_{3}O}_{10}^{3-}$) is predominantly tricharged under these conditions (see Figure 1b), which is sufficient for cross-linking of the amino groups of the chitosan polycation.

In the literature, the pH dependence of ChNP sizes has been studied in two ways. The first one involves adjusting the pH of the initial chitosan solution before adding TPP by varying the concentration of the acid used to dissolve Ch (increased pH) or by introducing an inorganic or organic base (decreased pH). Section 2.1 already analyzed the effect of pH, determined by varying the concentration of AcA, on nanoparticle sizes. In this regard, we will consider additional aspects that have not been covered previously.

Fan et al. [58] varied the pH of the initial ChNP solution in the range of 3.6–5.5 not only by varying *C*_AcA_ but also by adding 20% NaOH. The results showed that the critical Ch:TPP mass ratio required to form an opalescent ChNPs suspension, regardless of the *C*_AcA_ used, decreased with decreasing pH. With pH below 4.5, it was difficult to obtain nanoparticles with a unimodal size distribution, while the presence of microparticles in the suspension was inevitable with pH above 5.2. The pH range of 4.7–4.8 was chosen as the optimal condition for neutralizing the Ch solution with the alkali one, since in this range even an excess AcA amount (in the case of a high *C*_AcA_) only leads to an increase in the ionic strength of the dissolving medium.

This result is confirmed in Ref. [57], where the average diameter of LMW ChNPs increased from 50 up to 200 nm, and the PDI decreased from 0.4 down to 0.1 with an increase in pH from 3.5 up to 5.5 using 4 N (~16%) NaOH. Since pH also depends on the concentration of AcA (the Ch solvent), an increase in *C*_AcA_ [58] leads to a decrease in pH, size, and polydispersity index of the obtained ChNPs. The authors derived an equation for the particle diameter *d*:

$$d=2129.7-312.59A-678.54B-16.22C+18.3AB-0.38AC+4.13BC+30.57A^{2}+67.38B^{2}+0.07C^{2},$$

where *A* is the Ch:TPP mass ratio, *B* the pH of the Ch solution, and *C* the temperature (°C). This allows for guidance in the formation of nanoparticles with a pregiven size, including the smallest one. However, the plotted graphs and 3D surfaces provide information only on the average size of ChNPs, ignoring the quality of the size distribution and the presence of microparticles.

The pH of the ionotropic reagent solution has a significant impact on the size and polydispersity of ChNPs. Using a TPP solution with pH < 4.0 leads to incomplete cross-linking due to excessive protonation of both the Ch amino groups and the TPP phosphate groups. In solutions with pH > 6.5, deprotonation of the Ch amino groups occurs, resulting in weakening of ionic interactions with TPP and dissociation of the Ch–TPP complexes. The optimal pH for strong cross-linking of Ch macrochains corresponds to the range of 4.5–5.5, which is typically achieved using HCl [57,79]. Under such conditions, the amino groups of Ch remain partially protonated, providing sufficient positive charge for interaction with the negatively charged phosphate groups of TPP. Within this pH range, the latter are also in their optimal ionization state (${{H_{2}P}_{3}O}_{10}^{3-}$), (see Figure 1b and Figure 3) for efficient interaction with $\sim {-\mathrm{NH}}_{3}^{+}$. For LMW Ch samples, pH values up to 6.0–6.2 are also acceptable [86]. For example, Hashad et al. [87] demonstrated the possibility of obtaining nanoparticles by ionic gelation of Ch with TPP at pH 6.2 (prepared by adding 1 N {~4%} NaOH) with high process efficiency (ChNPs yield up to 91.5%). An LMW Ch sample with an MM of 50 kDa and DA = 75–85% was used. To prevent polymer precipitation at this pH, the variable concentrations and mass ratios of the components were optimized as follows: *C*_Ch_ = 0.1–0.3 g/dL, *C*_AcA_ = 1.0 g/dL, *C*_TPP_ = 0.01–0.05 g/dL, and the Ch:TPP ratio from 9:1 to 5:1. The authors note that at these concentrations/ratios, an optimal balance is achieved between the degree of protonation of the Ch amino groups and the degree of ionization of the TPP phosphate groups at pH = 6.2. The most optimal composition was characterized by the formation of ChNPs with a spherical compact morphology, an average hydrodynamic size of 227 nm, and a zeta potential of +24.3 mV.

Regarding the use of an aqueous TPP solution (i.e., with no addition of HCl) to obtain ChNPs, it should be taken into account that this solution has an alkaline reaction of the medium (pH ~9.7) due to hydrolysis, but it predominantly contains the fully deprotonated form ${P_{3}O}_{10}^{5-}$. This could theoretically provide an electrostatic interaction with the polycation, but the aminopolysaccharide is in a neutral form at such a high pH, which excludes the possibility of ionic cross-linking [40,88,89]. However, despite the insolubility of chitosan in an alkaline medium, upon slow addition of an aqueous TPP solution to a moderately acidic Ch solution, the hydroxide ions, which are present in smaller quantities, are quickly neutralized by the hydronium ions, which are in excess, which prevents undesirable effects on ~${-\mathrm{NH}}_{3}^{+}$ [58]. However, as titration progresses, when the [–NH_2_]/[PO_4_] ratio becomes small, neutralization of the acidic chitosan solution becomes noticeable (pH increases), although the Ch concentration in solution also decreases significantly due to the polymer’s transformation into nanoparticles. Thus, the effect of pH merges with that of the [–NH_2_]/[PO_4_] ratio, and one can only tentatively discuss the properties of the formed Ch at a specific pH, as this value changes during titration.

Another approach to the influence of pH on the size characteristics of ChNPs involves varying the acid–base properties of the dispersion medium of the resulting nanosuspension. López-León et al. [90] placed the prepared nanoparticles into a buffer medium of varying acidity and observed their size changes, which essentially represented an analysis of the nanogel swelling process. The particles were obtained from LMW Ch hydrochloride (Protasan 110 Cl) with *M*_n_ > 50 kDa and a DD of 87%. Two types of buffer solutions with an ionic strength of 2 mM were used, namely: anionic buffers based on acetate (pH 4 and 5), phosphate (pH 6 and 7), and borate (pH 8, 9, and 10) solutions; and cationic buffers to maintain pH 6 and 7 (Bis-Tris), pH 8 and 9 (Tris), and pH 10 (2-amino-2-methyl-1-propanol). With an increase in pH from 4 up to 7, the average diameter of ChNPs monotonically decreased from ~300 to ~200 nm (note that the p*K*_a_ of chitosan amino groups is 6.0–6.5), and with a further increase in pH up to 10, it remained constant. This is likely due to the remaining uncross-linked protonated amino groups, whose deprotonation with increasing pH reduces the repulsion of the positive charges of the glucosamine units of the macrochains and promotes deswelling (dehydration) of the ChNPs and greater compaction of the macrochains in their structure. Of course, the Ch macromolecules in the primary nanosuspension should not be excessively crosslinked. This behavior is confirmed by measuring the electrophoretic mobility of the nanoparticles and the isoelectric point, which for the studied Ch–TPP ionic complex was ~7.5. With prolonged exposure of ChNPs to buffer media with pH > 7, the mobility sign is inversed, and the nanosuspension becomes colloidally unstable due to the predominance of tripolyphosphate ionic groups in the nanoparticles, since the glucosamine groups of the polymer are electrically neutral at alkaline pHs.

The pH-dependent swelling of ChNPs in buffer media of varying acidity was also observed by Raj et al. [91]. Nanoparticles, as in the previous study [90], were obtained from LMW Ch (DD ≥ 95%, standard viscosity 20–300 cP) using the standard ionotropic gelation method, but were additionally lyophilized to obtain a nanostructured powder. Air-dried ChNPs had a uniform spherical shape (SEM), sizes of 161 ± 6.2 nm (DLS), a polydispersity index of 0.21 ± 0.02, and a zeta potential of 22 ± 2.5 mV. The swelling percentage of the obtained nanopowders in a phosphate buffer with pH 1.2, 6.8, and 7.4 was found to be 474 ± 24%, 196 ± 17%, and 152 ± 17%, respectively, over 24 h (unfortunately, the authors did not monitor the change in NP size during swelling). This sorption behavior is quite expected, given the higher solubility of ChNPs in acidic media.

A somewhat different behavior of ChNPs was observed in aqueous solutions of strong electrolyte salts. López-León et al. [90] found that varying the ionic strength of the nanosuspension dispersion medium by adding KCl led not only to initial swelling but also to subsequent degradation of the ChNPs and dissolution of their constituent components. This effect depended on both the salt concentration and pH. At low KCl concentrations (below ~30 mM at pH 4 and ~15 mM at pH 7), a reversible increase in the size of Ch–TPP nanoparticles occurred, most pronounced at neutral pH. At higher KCl concentrations, the swelling process was replaced by ChNP disintegration, and at pH 7, disintegration was observed at lower KCl concentrations than at pH 4. Disintegration of ionically cross-linked Ch–TPP complexes in 150 mM NaCl (pH 5.5) was also observed in Ref. [92]. The mechanism underlying this behavior of ChNPs in aqueous-salt solutions is that monovalent salt ions (K^+^, Na^+^, and Cl^−^) screen charges in the polymer network of nanogel particles (Debye screening due to an increase in the ionic strength of the solution and a denser ionic atmosphere), thereby weakening the intensity of the Ch–TPP ionic interaction and, consequently, the strength of cross-links. The obtained results are consistent with the theory of gel swelling under the action of salts in partially ionized polymer networks (the Flory–Huggins thermodynamic theory based on solvent–polymer interactions and elastic contributions of the network).

A comparative study of the stability of ChNPs in electrolyte salt solutions with controlled ionic strength by adding NaCl (pH 4–7), as well as in phosphate and phosphate-saline buffers (pH 7.2) [79] revealed dissolution of ChNPs in aqueous-salt solutions similar to the previous two studies. Furthermore, a significant and highly nontrivial effect of the degree of aminopolysaccharide deacetylation on the stability of ChNPs in the studied media was revealed, e.g., the dissolution ChNP stability increased with increasing chitosan DD, as this increased the number of –NH_2_ groups capable of protonation and subsequently ionic binding with TPP, forming strong cross-links resistant to dissociation. For example, Ch-based nanoparticles with a DD within 91–96% demonstrated high stability over the entire studied range of NaCl concentrations. As DD decreased down to 82%, partial dissolution of ChNPs was observed over time, and complete dissolution occurred at DD of 72–63%. The lower the DD of the sample, the lower the NaCl concentration required to initiate NP dissolution. At the same time, the aggregation stability of ChNPs under conditions close to pH 7–7.2 decreased with increasing the polymer’s DD due to its lyophobization as it approaches its isoelectric charge point. The obtained results are consistent with Ref. [81], which showed that the stability at pH 7.4 is higher for ChNPs obtained from chitosan with DD ≤ 73%, as well as with an earlier study [93], which showed that nanoparticles from Ch with a high DD exhibit stronger intermolecular interactions with TPP.

It is important to emphasize once again that if a monovalent salt-electrolyte is introduced into the initial Ch solution before TPP titration, the resulting ChNPs will exhibit higher kinetic stability compared to NPs obtained from Ch solutions without the addition of salt, despite a decrease in the ζ-potential, and are characterized by smaller sizes (see Section 2.3) [74,82]. However, the introduction of salt into the composition of ChNPs may negatively affect their biological activity. Few studies [40,86] also confirmed the importance of pH and ionic strength of the medium for the colloidal stability of ChNPs in suspension.

The lability of Ch–TPP ionic complexes in the ChNPs structure with dissociation into individual components was also observed when the acidity of the medium was reduced by increasing pH by introducing a strong base. E.g., suspensions of ChNPs obtained from a solution of Ch hydrochloride (Protasan UP Cl 214) with M_w_ 560 kDa, DD 93.2 ± 2.4% at pH 4.4 were brought to neutral and slightly alkaline pH levels (7.4 and 8.9, respectively) by adding 5% NaOH solution and analyzed to find their composition (Ch, TPP) [88]. At pH 7.4, free Ch chains and TPP residues were detected in the system, and at pH 8.9, phase separation occurred to form precipitated Ch forms. An explanation for this behavior of ChNPs is given (as well as in Refs [90,92]) from the position of a change in the charge of the polyions. With increasing pH, the degree of Ch protonation decreases, while the degree of TPP ionization increases, leading to the disruption of electrostatic interactions and dissociation of the Ch–TPP complex. These results confirm the importance of maintaining an acidic pH for the stability of ChNPs and should be considered when developing chitosan-based agrochemical nanostructures.

## 3. ChNP Size Distribution

The size distribution of ChNPs is a critical characteristic determining their physicochemical properties, kinetic stability, biological functionality, and, consequently, their effectiveness in practical applications. However, researchers have not studied this characteristic in sufficient detail and typically limit themselves to reporting the average size of nanoparticles in solution or in the air-dry state. The size distribution of cross-linked Ch–TPP nanoparticles, such as their average size and polydispersity index, depends on the MW and DD of the polymer, the concentration and ratio of reagents, the acidity of the reaction medium, the temperature of the ionotropic gelation reaction, and ultrasonic treatment.

**Figure 4:**
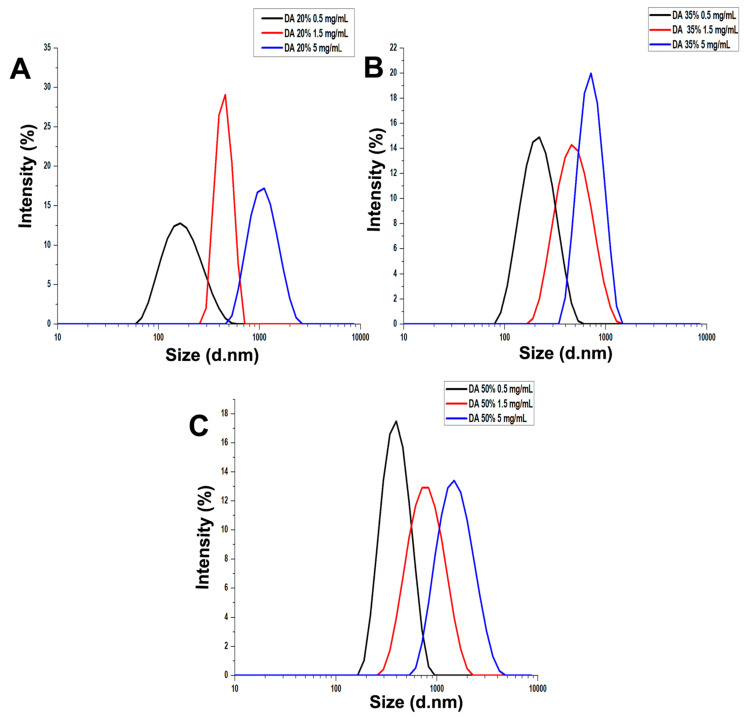
Size distribution of chitosan–TPP particles. Chitosan samples at different DA and concentrations (black 0.05 g/dL, red 0.15 g/dL, blue 0.5 g/dL): (**A**) DA 20% with a –NH_2_/PO_4_ ratio of 1.5; (**B**) DA 35%, –NH_2_/PO_4_ = 1; (**C**) DA 50%, –NH_2_/PO_4_ = 1.5 [56] (licensed under Creative Commons CC BY). Note: Author’s figure captions have been adapted for consistency.

For example, a systematic study conducted by Sreekumar et al. [56] showed that the size distribution of ChNPs is strongly dependent on both the concentration of Ch in the initial solution and the degree of its acetylation (unfortunately, the average polymer MWs also differed in this case) and, indirectly, on the Ch:TPP ratio (Figure 4). The distribution curves shift toward larger particle sizes with increasing *C*_Ch_ in the solution (the probability of binding of adjacent macromolecules increases) and with increasing DA of the polymer sample (for the same number of bound amino groups, determined by the amount of added TPP, there is a greater number of macromolecules). This confirms, but in more detail, the trend discussed in Section 2.1 and Section 2.2 on the influence of these parameters on the size characteristics of ChNPs, which allows us to conclude that the shape of the size distribution curves correlates with the molecular-compositional heterogeneity of the samples.

**Figure 5:**
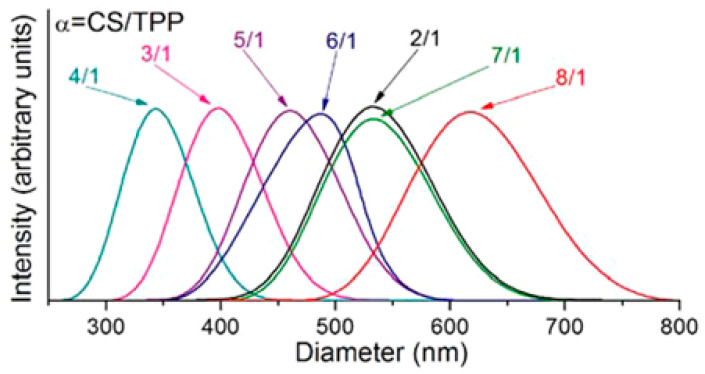
Size distribution of ChNPs depending on the Ch/TPP ratio [40] (licensed under Creative Commons CC BY).

The size distribution of ChNPs as a function of the polymer:ionotropic agent ratio is also of interest, since the effect of the agent is due to the mechanism of nanoparticle formation based on the electrostatic interaction of the chitosan polycation with TPP anions. For example, Koukaras et al. [40] obtained ChNPs in the size range of 340–615 nm, so the trend discussed in Section 2.3 regarding the existence of an optimal Ch:TPP ratio for each specific case, in this case 4:1 (Figure 5), is confirmed. With an optimized polymer:ionotropic agent composition, a balance is achieved between a sufficient amount of TPP anions for effective cross-linking and the prevention of excessive cross-linking, which allows for the production of nanoparticles with a narrow size distribution and a polydispersity index of <0.3 [78]. With deviations from this optimal ratio, the PDI increases to 0.4–0.5 and higher. Moreover, at non-optimal Ch:TPP ratios, a bimodal size distribution may be observed, which is associated with the simultaneous formation of primary nanoparticles and their aggregates [94].

The polydispersity of chitosan nanoparticles is also affected by the average molecular weight of the polymer. LMW Ch promotes the formation of more monodisperse nanoparticles [58], while HMW one forms more polydisperse ones [77]. At the same time, Ing et al. [68] obtained ChNPs with a relatively narrow size distribution (PDI 0.4–0.6) for LMW and HMW Ch. For the HMW sample, this was achieved by decreasing the initial polymer solution concentration and maintaining its pH at 5.6. Moreover, the nanoparticle sizes of both Ch samples increased with increasing MW.

According to Ref. [58], the size distribution of ChNPs can be significantly narrowed by simultaneously decreasing the AcA concentration and the reaction temperature of ionotropic cross-linking. The authors succeeded in obtaining monodisperse nanoparticles with an average hydrodynamic diameter of 130–140 nm and a polydispersity index of less than 0.05. Abdel-Hafez et al. [57] proposed statistical methods for designing experiments to optimize the size distribution of Ch nanoparticles. Their statistical calculations were based on a large array of experimental data on the production of ChNPs with diameters within 52–400 nm and PDIs within 0.06–0.40 by varying *C*_AcA_ and *C*_TPP_, the pH of the polymer solution and the cross-linking reagent, the Ch:TPP ratio, temperature, and stirring speed. It was found that the response surface construction methods (Box–Behnken and D-optimal) allowed predicting the particle size with high accuracy (percentage deviation of only 1.5%). As for the air-dried state of ChNPs, the use of a cryoprotectant (D-trehalose) and ultrasonic treatment to prevent agglomeration of nanostructures during freeze-drying was proposed to optimize their average size and size distribution and to increase stability [95].

It should be noted that the initial chitosan samples (both LMW and HMW) were characterized by a certain molecular weight distribution (MWD). However, in all the abovementioned studies, the authors only report the average molecular weight Ch, as provided in the sample datasheet or determined in the laboratory. Given the revealed regularities of influence of the physicochemical characteristics of the aminopolysaccharide (MM, DD/DA) on the polydispersity of the resulting ChNPs, it can be assumed that the size distribution of the nanoparticles reflects the MWD of chitosan. Of course, this is taking into account that one nanoparticle could contain few macromolecules with different MWs. In any case, since the authors of different papers do not correlate the size distribution of their ChNPs with the molecular weight distribution of the macromolecules in the sample used and do not study their possible correlation, this hypothesis remains speculative, and detailed, comprehensive experiments are needed to confirm it.

## 4. Zeta Potential of ChNPs

Zeta potential is an important key parameter reflecting the total surface charge of ChNPs. This quantity determines the aggregation and sedimentation stability of ChNPs in suspension, as well as their behavior under various conditions, including interactions with plant objects, microorganisms, phytopathogens, etc. Our analysis of literary sources has shown that the zeta potential of ChNPs (including those for agrobiochemical applications), as well as their average size and size distribution, significantly depends on the MW and DD/DA of the aminopolysaccharide used, the concentration and ratio of reactants, the pH and ionic strength of the reaction medium, the gelation time, and the storage conditions of the finished nanoparticles.

It was found that the zeta potential of ChNPs increases in the range from +22 to +55 mV depending on the concentration and molecular weight of the polymer [68]. In the first case, each TPP anion cross-links a larger number of Ch macromolecules located in close proximity to each other, while in the second case, it cross-links longer macromolecules. In both cases, with an increase in *C*_Ch_ and MW, there are more amino groups per cross-linking reagent anion, and therefore per nanoparticle, some of which being positively charged. In addition, *C*_Ch_ and the MW of Ch samples may affect their ability to form intramolecular bonds due to differences in the conformation of polymer chains in solution, which also affects the electrical potential. An increase in the ζ-potential of ChNPs with an increase in *C*_Ch_ was also established by Fan et al. [58], and with an increase in both *C*_Ch_ and MW—in two works [70,96]. In addition to the above, it has been shown that nanoparticles based on LMW chitosan achieve stable zeta potential values during their production process faster compared to HMW analogs [58].

The influence of the degree of acetylation/deacetylation of chitosan on the ζ-potential and, as shown in the previous subsections, on the physical sizes, polydispersity index, and the yield of the formed nanoparticles was also found. With an increase in DD (a decrease in DA), the number of free amino groups capable of protonation and, accordingly, interaction with TPP increases [40,77,79,81]. Therefore, for Ch samples with a higher DD, a larger amount of TPP is required for charge neutralization compared to chitosan samples with a lower DD. Highly acetylated chitosan was also found to exhibit a lower sensitivity of its zeta potential to the physicochemical parameters of the ionotropic cross-linking reaction [77], e.g., systems based on Ch with the lowest degree of acetylation (DA ~ 0%) show higher zeta potential values of +55–+60 mV, which are practically independent of *C*_Ch_[η]. With an increase in DA to 22 and up 35–47%, the zeta potential of ChNPs decreased to +30–+40 and +15–+18 mV, respectively. This character of the electrokinetic parameter of NPs depending on the DA of the samples is due to a change in the proportion of hydrophilic/hydrophobic fragments in the composition of macromolecules and, as a consequence, their flexibility. It is important to note that a lower DA (a higher DD) increases the solubility of Ch in an aqueous-acidic medium and, accordingly, the number of protonated amino groups, which increase the positive charge of the nanoparticles, but reduces their kinetic stability [79].

**Figure 6:**
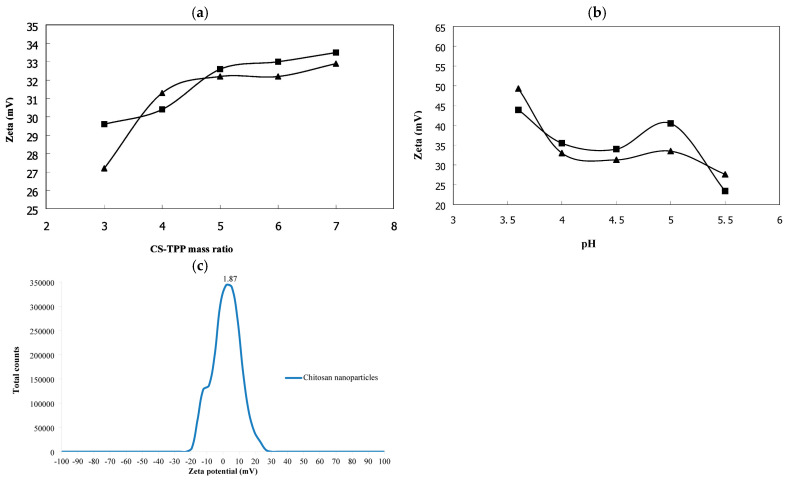
Effect of ionotropic gelation reaction conditions on the ζ-potential of ChNPs. (**a**,**b**)—Effect of Ch:TPP weight ratio (**a**) and initial pH of the Ch solution (**b**) on the ζ-potential of ChNPs; MW Ch 50 kDa, *C*_Ch_ 0.05 (▲) and 0.15 (■) g/dL [70] (licensed under Creative Commons CC BY). (**c**)—Distribution of ChNPs by zeta potential [99] (licensed under Creative Commons CC BY). Note: Author’s figure legends have been adapted for unification.

Along with the concentration, molecular weight, and degree of deacetylation of Ch, the concentration of the cross-linking reagent and its ratio to the aminopolysaccharide are effective parameters for controlled changes in the surface charge of ChNPs. An increase in *C*_TPP_ or a decrease in CS:TPP leads to a monotonic decrease in the zeta potential of ChNPs, which is explained by neutralization of the positive charges of the amino groups of Ch by the negative phosphate groups of TPP [79,82,89]. The optimal Ch:TPP value provides a sufficient number of positive charges to form stable nanoparticles [97,98]. For example, a systematic study of the effect of the CS:TPP mass ratio (at *C*_Ch_ = const) on the properties of ChNPs [89] showed a decrease in the ζ-potential from ~+45 mV down to ~+15 mV with an increase in the TPP content. Fan et al. [58] found that the zeta potential (probably of the aggregate of cross-linked ChNPs and non-cross-linked Ch macromolecules) decreased almost linearly from +39 down to +26 mV with increasing volume of the TPP solution added. Hu et al. [70] similarly noted that the ζ-potential dropped sharply with decreasing Ch:TPP ratio from 5:1 down to 3:1, but with an increase up to 7:1, some increase in the surface charge was observed, which may be due to lesser neutralization of protonated amino groups along with weakening of ionic binding (Figure 6a). Antoniou et al. [82] also found that the ζ-potential of NPs at low Ch:TPP values decreased significantly. At the same time, at a Ch:TPP ratio of 3:1, extremely intense aggregation of particles was recorded, accompanied by rapid precipitation of the polymer. At very high ratios of the reacting components (Ch:TPP > 7:1), the achieved ζ-potential values of ChNPs remained virtually constant (although the particle size increased).

Thus, as the TPP concentration in the reaction mixture increases, partial or complete compensation of the surface charge occurs, which leads to a decrease in the ζ-potential of ChNPs and a change in the colloidal stability of the nanosuspension [79,82,97]. At a certain CS:TPP ratio, depending on the MW and DD of chitosan, it is possible to achieve conditions close to the isoelectric point of the polyelectrolyte, with the ζ-potential tending to zero. As a result, a further increase in *C*_TPP_ may lead to a recharging of the particles and the appearance of a negative ζ-potential [100,101,102,103]. The negative ζ-potential values may vary in the range from −3 to −37 mV (Table A1). In some cases, a fairly wide distribution of ChNPs by ζ-potential was observed, covering both the positive (up to +15–+30 mV) and negative (down to −20 mV) regions [99,104]. Unfortunately, insufficient attention was paid to the explanation of the negative value of this quantity, which complicates the correct understanding and analysis of the obtained results. Let us cite Ref. [99] as an example (Figure 6c). The authors do not discuss the variation in the electrokinetic potential in the range of positive and negative values, limiting themselves to providing an average value of ~1.9 mV (indicated at the maximum on the corresponding graph). However, such a low value indicates, firstly, the low aggregative stability of the obtained ChNPs, and, secondly, the almost complete absence of protonated amino groups, i.e., the poor biological activity of such nanoparticles (the same applies to the left half of the distribution, negative values of the ζ-potential). One way or another, complete charge inversion, leading to negative values of the electrokinetic potential, is achieved comparatively rarely in the classical ionotropic gelation reaction using TPP. Salts of hexaphosphoric acid, which have a longer chain length and a more negative charge than TPP (e.g., sodium hexametaphosphate or metaphosphate), and the reverse order of mixing the reagents (introduction of a Ch solution into a polyphosphate solution) are generally required to obtain negatively charged ChNPs with *d* = 100–200 nm and a narrow ζ-potential distribution [105]. With an excess of sodium polyphosphate, the particles acquire a negative charge due to the shielding of the positively charged amino groups of the polysaccharide chain by excessive negative phosphate groups.

Based on our literature analysis, the following generalized patterns of the influence of the ratio of reactants in the ionotropic cross-linking reaction on the electrokinetic potential of ChNPs can be presented [77,82,90,97,98]. At a mass ratio of CS:TPP ~ 3:1–6:1, a high positive ζ-potential (+30–+45 mV) and high colloidal stability are realized; at CS:TPP ~ 2:1 and below, a moderate positive ζ-potential (+10–+30 mV) and moderate colloidal stability are realized. At a CS:TPP ratio ~ 1:1, low positive or zero values of the ζ-potential are observed, and potential inversion to negative values and variable aggregation and sedimentation stability of ChNPs are possible at a very high *C*_TPP_. The typical CS:TPP ratio, which provides an optimal balance between zeta potential and colloidal stability, as well as particle size, ranges from 3:1 to 6:1.

The zeta potential is also affected by the reaction time of ionotropic gelation, since the interaction between $-{\mathrm{NH}}_{3}^{+}$ and ${{H_{2}P}_{3}O}_{10}^{3-}$ occurs gradually [40]. At the initial stage of TPP introduction into an aqueous-acidic CS solution, particles with a highly positive charge are formed, since the formation of ionotropic bonds occurs predominantly on the surface of the polymer coils [79]. Over time (~30 min), the cross-linking process extends deeper into the particles, which is accompanied by a reorientation of the macrochains, a change in the charge distribution, and, consequently, the zeta potential of ChNPs [77]. In the subsequent time range (30 min—2 h), as the main ionic binding reactions are completed, additional cross-linking, charge redistribution within the particles, and gradual stabilization of the potential proceed [58,90,106]. During prolonged gelation (>2 h), minor changes in the ζ-potential can be observed, caused by slow relaxation processes of the polymer networks and possible rearrangement of the particle structure [107]. It is also noted that increased concentrations of components in the inotropic cross-linking reaction contribute to the acceleration of gelation processes and the more rapid achievement of stable ζ-potential values [90].

The zeta potential of ChNPs is also determined by the initial pH of the chitosan solution, since the latter significantly affects the degree of amino group protonation [106]. The ζ(pH) dependence obtained in Ref. [70] for solutions with *C*_Ch_ = 0.05 and 0.15 g/dL (Ch MW 50 kDa, Ch:TPP = 5:1) shows that the lowest ζ-potential of ChNPs is achieved at pH 4.5 for both polymer concentrations (Figure 6b). Below pH 4.0, strong protonation of –NH_2_ groups led to a higher ζ-potential and stronger intermolecular repulsion, causing the polymer chains to stretch and form larger ChNPs. Protonation of –NH_2_ groups at pH 5.5 was so weakened that the ζ-potential dropped sharply. A similar dependence is given in Ref. [82], where characteristics and concentrations of Ch similar to those in the previous work were used (MW 100 kDa, DD 90%, *C*_Ch_ = 0.05 and 0.15 g/dL). A nearly linear decrease in the ζ-potential of ChNPs with increasing pH was found for both *C*_Ch_.

As discussed in the previous sections, a strong monovalent salt (usually NaCl) is added to the Ch solution in some cases to obtain colloidally stable ChNPs with *d* < 100 nm, which significantly affects the ζ-potential of the resulting nanoparticles [74,82]. For example, Antoniou et al. [82] showed that the addition of NaCl (0.05–0.3 g/dL) significantly decreased the ζ-potential of ChNPs at all initial Ch concentrations used (0.05, 0.15, and 0.3 g/dL). The addition of 0.3 g/dL NaCl led to a decrease in the ζ-potential from +18.5 to +15.5 mV when a Ch solution with a concentration of 0.15 g/dL is used to obtain ChNPs. The most pronounced effect was observed for *C*_NaCl_ = 0.1 g/dL and *C*_Ch_ = 0.05 g/dL, i.e., a decrease from 31 down to 20 mV. The introduction of a strong electrolyte salt weakens the interaction between Ch and TPP due to the neutralization (screening) of the charge on the chitosan chains. In addition, NaCl addition expands the concentration range of Ch in which NPs can be obtained, ensures a narrow size distribution of ChNPs, and improves the colloidal stability of the nanosuspension. For example, NPs obtained with NaCl addition are characterized not only by a smaller size and ζ-potential, but also exhibit the best kinetic stability during storage. In particular, the ζ-potential of such ChNPs did not change over the course of a month and remained at the level achieved 24 h after their preparation [74]. Nevertheless, longer storage may lead to changes in the surface properties of NPs due to relaxation of polymer chains or additional interparticle interactions. Furthermore, despite the excellent properties of ChNPs obtained in an aqueous, acidic, and salty environment, excess salt (when such nanobiopreparations enter the environment) may disrupt plant physiological processes and reduce soil fertility.

Thus, the zeta potential values of ChNPs are usually positive because the positively charged amino groups of chitosan predominate over the negatively charged tripolyphosphate anions, as the cross-linking is partial or incomplete (Table A1). The optimal range of values is considered to be +25–+35 mV (see, for example, Refs [58,68,71,99,100,102,108,109,110,111]), which ensures colloidal stability of nanoparticles in an aqueous environment and their ability to interact with negatively charged substrates of phytopathogens. As the positive ζ-potential decreases, the stability of nanoparticles in the system decreases as well. High positive values, more than +50–+70 mV, are undesirable due to an insufficient cross-linking degree of macrocoils. Nanoparticles with a negative ζ-potential have been little studied so far [99,100,101,102,103,104]. Nevertheless, there is a possibility that they have significant practical potential and could effectively interact with cellular structures through non-electrostatic mechanisms such as hydrophobic interactions, chelation of metal ions, etc.

## 5. Conclusions

Our analysis of the research literature devoted to the production of ChNPs by ionotropic TPP cross-linking has revealed the high demand for this method in scientific work (155 cited works in the reference list). The general methodological approach involves cross-linking Ch macromolecules using ${{Na}_{5}P}_{3}O_{10}$ by ionotropic gelation in aqueous-acidic solutions under mild conditions. Diluted acetic acid (pH 4.5–5.5) is mainly used to dissolve ChNPs, less often an acetate buffer (pH ~5.0), and in some cases, biologically active organic acids such as ascorbic or citric acids are used. The ChNP solution concentration, depending on the physicochemical characteristics of the polymer, varies in the range of 0.025–1.0 g/dL. The cross-linking reagent is dissolved in water, and the acidity of the medium is typically maintained at the pH of the ChNP solution. TPP, forming multiply charged anions (${{H_{2}P}_{3}O}_{10}^{3-}$ and ${{H_{3}P}_{3}O}_{10}^{2-}$) in this pH range, electrostatically binds protonated amino groups ($-{\mathrm{NH}}_{3}^{+}$) of the polysaccharide (which are known to be necessary for performing various functional biological tasks), forming a gel complex as nanoparticles with a size of ~20 nm in a colloidal system. To obtain a stable opalescent suspension, without possible intense aggregation of nanoparticles, the vast majority of studies use TPP solutions with concentrations within 0.01–1.0% and CS:TPP ratios within 3:1–5:1. With an excess of the ionotropic cross-linker, a decrease in the ζ-potential values is observed, accompanied by a significant decrease in the aggregation and sedimentation stability of ChNPs.

In few papers [57,71], the authors use mathematical methods of experimental design, obtaining algebraic equations, to optimize the resulting ChNPs for various parameters. Unfortunately, no dependence of the coefficients in these equations on other properties of Ch samples and experimental conditions was established, which limits the value and versatility of the obtained dependencies. In this regard, a need arises for a comprehensive study involving Ch samples with a wide range of cross-linking characteristics and conditions.

It is also worth emphasizing that the criteria for the properties and functional qualities of the resulting ChNPs are ambiguous. Researchers are certainly striving to obtain nanoparticles with a minimal size (*d* ≤ 100 nm) and maximum positive ζ-potential. In addition, potentially practical properties of ChNPs are being tested, based on the encapsulation of agrochemicals and other biologically active substances within their structure, to develop methods for protecting agricultural crops from pests, stimulating plant growth and increasing their resistance to stress factors, and solving other agrobiotechnological problems [23]. However, given that different publications analyze different properties and qualities of ChNPs, conducting an accurate comparative analysis is not always possible.

In our opinion, some experimental conditions affecting the properties and quality of ChNPs are not properly taken into account, e.g., the stirring rate during ionic gelation significantly affects the reaction yield [61] and, therefore, by manipulating this parameter, it is possible to influence the morphology, size, electrochemical and other characteristics of the resulting nanoparticles [57,58]. However, the authors of most studies limit themselves to providing only the value of the stirring rate of the reaction mixture, without justifying the choice or varying possible options. Meanwhile, the stirring intensity determines the hydrodynamic conditions in the reaction medium, which directly affects the nucleation and growth processes of nanoparticles, e.g., the stirring rate controls the rate of mass transfer of reagents and the uniformity of their distribution inside the volume of the reaction system, the efficiency of dispersion of droplets of TPP solution in the Ch solution, the degree of cross-linking of macrochains, as well as shear stress affecting the forming cross-linked polymer structures. Moreover, the influence of stirring intensity is not an isolated parameter but correlates with such physicochemical characteristics as the concentration and molecular weight of chitosan, pH of the medium, and temperature [40,57,58,61]. Under certain optimal conditions for ionotropic gelation, all these factors could reinforce each other, resulting in synergistic effects.

The influence of the chitosan solution viscosity and the crossover concentration, which are often not measured or discussed, are also underestimated, with the exception of Refs [56,77]. The application of the approach proposed in these studies by other authors using a wide range of Ch samples with different physicochemical parameters could provide additional information for obtaining ChNPs with optimal characteristics and properties (minimum diameter, narrow size distribution, zeta potential sufficient for colloidal stability, etc.).

In general, in the vast majority of cases, the factors studied by the authors (reagent concentrations and their ratios, pH, temperature, ionic strength, degree of Ch deacetylation) relate to equilibrium. However, the process of obtaining ChNPs is characterized by significant deviations from equilibrium, which requires a more thorough analysis of the kinetic factors of the ion gelation process. Therefore, understanding the mechanisms by which thermodynamic (reagent concentrations, pH, temperature, ionic strength) and kinetic factors (addition rate and volume of cross-linking agent, stirring speed, stirring duration after addition of all TPP, suspension holding time before ChNPs are released, cooling rate) influence nanoparticle formation is key to optimizing the production processes and improving the functional properties of nanostructured chitosan materials.

Since TPP acidification with HCl is recommended, it might seem that residual chloride could have negative effects on ChNP-based agrobiochemicals. But an acidic medium is always used to dissolve chitosan, so an acid anion (chloride, acetate, or another) will always be present, including within the liquid absorbed by the nanogel. However, in most cases, the resulting nanoparticles are separated and purified, which removes most of the impurities. In any case, the amount of chloride (as well as acetate) is unlikely to pose any chemical hazard.

There are challenges related to scale-up, commercialization, regulatory requirements, and environmental safety, but they are rarely addressed by the authors of research papers. For example, ultra-pure and therefore expensive reagents are usually purchased from Sigma Aldrich for research, but where will reagents for large-scale production be sourced? Much work remains to be done in this area. We encourage future authors to not neglect these issues.

## 6. Future Directions

**Figure 7:**
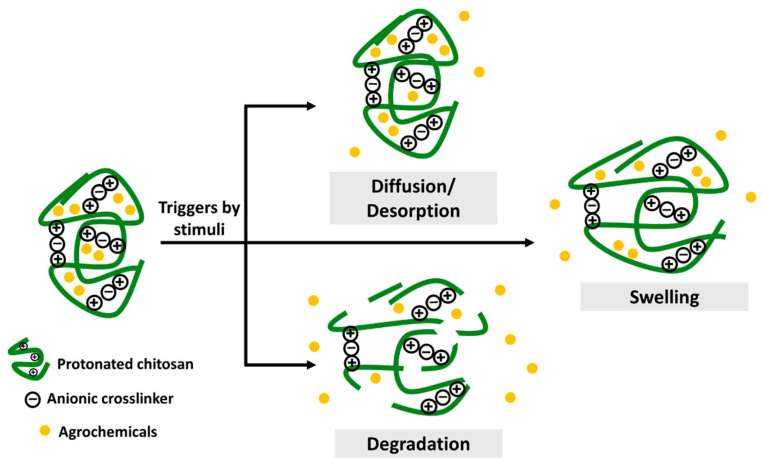
Release mechanism of active substances from chitosan-based agronanochemicals [23] (licensed under Creative Commons CC BY).

Despite the widespread use of the ionotropic gelation method using TPP to obtain ChNPs, its significant drawbacks include the initial partial deprotonation of the aminopolysaccharide to compress the macrocoils, as well as the exclusively polyanionic nature of the cross-linking particle. First, since the key factor in the biological activity of chitosan is its cationic nature at pH < 6.0–6.5, raising the pH of the initial Ch solution by introducing an alkaline agent reduces the positive charge on the macrochains and, accordingly, deteriorates the biofunctionality of the resulting preparations [30,31,32,33]. Second, one TPP anion, binding to two protonated amino groups of the Ch polycation (remaining after NaOH introduction) for cross-linking, deactivates them due to the formation of weakly charged Ch–TPP ionic complexes, which also removes the $-{\mathrm{NH}}_{3}^{+}$ groups from their functional biological role. Meanwhile, the major mechanism of biological activity of Ch, for example, its antimicrobial effect on phytopathogens (bacteria, fungi and viruses), is based on the electrostatic interaction of the polycation with the negative charge of the surface of cellular structures, accompanied by an increase in membrane permeability and subsequently cell death [31,112,113,114]. Chitosan deprotonation with additional ionic cross-linking of the remaining positively charged amino groups by negatively charged phosphate groups of TPP and, as a consequence, a decrease in the biological activity inherent in its protonated form, necessitate the incorporation of active ingredients into ChNPs (Figure 7) [23]. Table A1 shows that various fungicides (protocatechuic acid, saponin, Cu^2+^, Ag, hexaconazole), antibacterial agents and substances (Ag^+^, Cu^2+^, Zn^2+^, Mn^2+^, Fe^2+^, *Achillea millefolium* extract), insecticides (neem oil, karanj oil, azadirachtin and karanjin), plant growth stimulants (gibberellic and salicylic acids), and anti-stress agents (S-nitroso-mercaptosuccinic acid—against salinity stress) are introduced into ChNPs.

Based on the above, two possible approaches to producing ChNPs with high practical and functional significance for agrobiotechnology appear to be relevant, each with its own unique advantages and potential applications. In our opinion, it is advisable to focus on the use of biologically active organic acids and amino acids for dissolving chitosan, i.e., obtaining working solutions to form cross-linked nanostructures. Acids can play a dual role in this regard. While H^+^ creates an acidic environment and protonates the amino groups of Ch, ensuring its solubility in water, biologically active anions could impart specific target properties to the preparation, including increased biological activity [115,116,117,118]. For example, *Bombyx mori* chitosan ascorbate nanoparticles obtained by ionotropic cross-linking of macrochains of the salt form of Ch and ascorbic acid using TPP demonstrate a significantly higher fungicidal effect in vitro on *F. oxysporum* compared to the triazole fungicide tebuconazole [119]. The fungicidal activity in vitro and in vivo of chitosan ascorbate nanoparticles formed by a similar method against *Harpophora maydis* significantly exceeds that of the systemic fungicide triticonazole [120]. The ionotropic gelation of salt chitosan with TPP anions also produces stable compact ChNPs with a high surface charge based on salt complexes of Ch with amino acids (glutamic, aspartic) and alpha-hydroxy acids (lactic, glycolic) [121]. The use of dibasic amino acids or polybasic organic acids, such as citric acid, as cross-linking agents appears equally promising. The carboxyl groups of these acids, instead of the phosphate groups of TPP, could cross-link chitosan macromolecules at protonated amino groups, while their own amino group would remain free and could partially perform the functional role of the deactivated amino groups of Ch. It should be noted that Pilon et al. [122] used citric acid to dissolve chitosan, but only to ensure comparability under “all other things being equal” when comparing the efficiency of ChNPs and a usual chitosan gel coating in apple storage experiments. Citric acid was chosen because its use is permitted in the food industry, but it was TPP which served as the cross-linking agent to produce nanoparticles.

However, finding ways to obtain ChNPs without using any cross-linking agent at all seems most promising. For example, we have previously shown that the chitosan-aspartic acid salt complex undergoes phase segregation of the polymer substance at a nanoparticle level due to the counterionic association of the acid residue ions on the protonated polymer chains [123,124]. Supramolecular ionic nanoassociates of chitosan aspartate are characterized by a developed system of hydrogen bonds, as well as specific ion–dipole and Coulomb interactions [125,126]. This makes it possible to obtain polycationic ChNps of spherical shape with an average diameter of 50–80 nm, low polydispersity and high charge density with no use of any ionic cross-linking agent and with the preservation of the maximum protonated amino groups realized under the given conditions. Such ChNPs have been established to be non-toxic, to exhibit biocidal activity against both Gram-negative and Gram-positive microorganisms [123,127]. Due to the fact that such self-organizing systems are kinetically unstable, nanoparticle functionalization with a polysiloxane shell coating is possible to impart aggregation and sedimentation stability. It seems promising to use not traditional sol–gel precursors, in particular, the widely used tetraethoxysilane or other similar alkoxysilanes, but biologically active ones, for example, silicon tetraglycerolate [128]. The optimal conditions for obtaining shelled polycationic chitosan nanoparticles are still unknown and remain to be studied, as does the behavior of such particles under real field agrobiochemical conditions. However, it has already been established that such particles are colloidally stable and show no signs of phase separation for at least two years of storage at 4 °C, and also have antifungal activity against a wide range of soil-dwelling saprotrophic and phytopathogenic fungi [129], and to exert a high growth-stimulating effect on a number of grain, legume, and vegetable crops [130] without introducing the corresponding agrochemicals into their structure.

Based on the above, it can be concluded that the size parameters, zeta potential, aggregation stability, and biological functionality of ionically cross-linked ChNPs may vary significantly depending on the molecular weight of chitosan, the ratio of N-glucosamine/N-acetylglucosamine units in the macrochains, and the parameters of the ionotropic gelation process. For the targeted production of ChNPs with specified morphology, size, and properties, it is important to optimize the formulation for their production relative to the physicochemical parameters of the aminopolysaccharide, and for those with high biological activity, to use biologically active organic acids or amino acids to dissolve chitosan, and also to strive to preserve the maximum number of protonated amino groups responsible for the biological activity of nanostructured chitosan-containing preparations.

## Data Availability

No new data were created in this study.

## Supplementary Materials

The following supporting information can be downloaded at: https://www.mdpi.com/article/10.3390/polym18131668/s1, Table S1: Recent review articles on chitosan nanobiochemicals; Table S2: Comparison of different ChNP preparation methods.

## Abbreviations

The following abbreviations are used in this manuscript:

AcA	acetic acid
AFM	atomic force microscopy
Ch	chitosan
ChNPs	chitosan nanoparticles
DA	degree of acetylation
DCS	differential centrifugal sedimentation
DD	degree of deacetylation
DLS	dynamic light scattering
GE	garlic extract
HMW	high-molecular-weight
LD	laser diffraction
LMW	low-molecular-weight
M_n_	number-average molecular weight
MW	molecular weight
M_w_	weight-average molecular weight
MWD	molecular weight distribution
NPs	nanoparticles
PDI	polydispersity index
SEM	scanning electron microscopy
TEM	transmission electron microscopy
TPP	(sodium) tripolyphosphate
XRPD	X-ray powder diffraction

## Appendix A

**Table A1:** Characteristics of chitosan, reagents for the ionotropic gelation reaction, and chitosan nanoparticles obtained by ionotropic cross-linking with sodium tripolyphosphate.

Source	Ch Characteristics		Concentration of Reagents, g/dL ^a^			Volume of Reagents, mL, or Volume Ratio		pH	Characteristics of ChNPs ^b^				Encapsulated Reagent
	MW, kDa	DD, %	*C*_Ch_	*C*_AcA_	*C*_TPP_	*V*_Ch_	*V*_TPP_		Mean Size *d*, nm	Range of *d*, nm	ζ-Potential, mV	PDI	
[75]	–	–	0.1 0.2 0.3 0.4 0.5	1.0	1.0	–	–	–	19 ± 2.1 ^X^ 21 ± 2.4 ^X^ 120 ± 4.1 ^X^ 153 ± 4.4 ^X^ 165 ± 5.8 ^X^	–	–	–	—
[67]	150	90	0.5	0.1	0.25	6	2	–	–	25–30 ^T^	+49–+52	–	Protocatechuic acid
[66]	100–300	–	0.2	1.0	0.06	–	10	5.5	25.7 ^T^ 32.7 ^D^	–	+45.4		NPK fertilizer
[131]	190–370	≥75	0.1	1.0	0.25	1:1		–	29.9 ± 1.4 ^T^	–	–	–	CuO NPs
[132]	20–30 cps ^c^	85–95	0.1	1.0	0.1	50	50	–	–	32–73 ^D^	–	0.28	Pesticide PONNEEM^®^ (neem oil, karanj oil, azadirachtin and karanjin)
[133]	–	87.4	0.26	1.05	0.06	3:1		–	39 ± 18 ^D^	–	+17.7 ± 0.1	0.3 ± 0.01	S-nitroso-mercaptosuccinic acid
[64]	220	85	0.5	1.0	0.25	3	1	–	40 ^D^	28–49 ^D^	+51	<1	Cu
[134]	–	95%	0.5	1.0	0.25	3	1	4.6–4.8	–	40–50 ^S^	–	–	—
[135]	LMW&HMW	–	–	1.0	1.0	100	10	–	50 ^T^	40–70 ^D^	+48	–	—
[136]	–	–	–	1.0	1.0	100	10	–	50 ^T^	–	+20	–	Cu
[137]	– From phototropic nauplii		–	–	–	4:1		–	–	50–70 ^T^	–	–	—
[119]	101 *Bombyx mori*	–	3.6	0.9 ^d^	0.5	1:0.25		–	–	50–350 ^A^	–	–	–
						1:0.5				50–200 ^A^			
[69]	150	90	0.3	1.0	1.0	25	1	–	54 ^D^		+51.4	1.0	Ag^+^, Cu^2+^, Zn^2+^, Mn^2+^, Fe^2+^
[73]	70 190 684	75–85	0.3	1.0	0.3	–	–	4.6	60 ± 5.5 ^D^ 78.5 ± 6.8 ^D^ 105.2 ± 8.6 ^D^	–	–	–	—
[120]	–	–	0.2	1.0 ^d^	0.27	–	1	–	–	65–80 ^S^ 100–156 ^D^	–	–	—
[111]	190–370	≥75	0.1	1.0	0.25	–	–	–	~80 ^D^	–	+30	–	—
[55]	190–310	≥75	Made by Candelo Biotech SL (Albacete, Spain)						~80 ^S^ 251.1 ± 3.3 ^D^	–	+43.6 ± 0.1	0.3	Garlic extract
[103]	–	–	0.5	1.0	0.25	3:1		5	83.3 ^D^	20–50 ^T^	−28	0.31	—
[138]	–	–	0.5	1.0	0.25	3	1	4.6	90 ± 5 ^T^	40–180 ^D^	–	–	—
[71]	1129	≥75	0.1	1.0	0.08	100	25	4.6–4.8	100 ^D^	–	+35.3	–	Hexaconazole
[139]	–	–	0.1	1.0	1.0	10	5	–	<100 ^S^ 118 ^D^	–	–	–	*Achillea millefolium* extract
[56]	125–450	40–98.5	0.05–0.5	5% molar excess	0.5	3:1		–	–	100–1200 ^D^	–	0.1–0.4	—
[68]	70	75–85	0.1 0.2 0.3	2.0	0.1	3	1.2	–	101 ± 9.6 ^D^ 169 ± 13.5 ^D^ 348 ± 35.7 ^D^	–	+35 ± 6.5 +43 ± 2.1 +47 ± 4.4	0.4 0.5 0.6	—
	310	85	0.1 0.2 0.3						136 ± 8.6 ^D^ 276 ± 46.8 ^D^ 1265 ± 206.5 ^D^		+38 ± 1.7 +50 ± 1.8 +55 ± 3.5	0.4 0.8 1.0	
[102]	–	–	0.25	0.5	0.2	5:1		–	105 ^D^	–	−32.2	0.14	—
[122]	71.3	94	–	0.2 ^e^ 0.4 ^e^	–	70	28	–	110 ^D^ 300 ^D^	–	–	–	—
[140]	200	–	0.5	0.5	0.5	10:3			111 ± 21 ^D^	–	–	–	—
[141]	HMW	85–89	0.25	1.0	0.075	–	–	4.7	121 ^D^	–	–	0.4	—
[104]	27	85	0.1–0.3	0.06	–	–	–	–	122 ^D^	10–30 ^T^	−20–+15	0.36	Guar gum
[142]	376	69	0.1	0.15	0.05	30	20	–	150 ^D^	–	–	–	Indole-3-acetic acid
[63]	– LMW	–	0.175	pH 4 ^f^	0.1	4:1		–	150 ^T^ 210 ^D^	–	+40	–	—
[65]	200–500	91.8	0.025 0.05 0.075 0.10	–	–	3:1–7:1		–	152 ± 1 ^D^ 232 ± 5 ^D^ 307 ± 4 ^D^ 377 ± 6 ^D^	–	+38–+43	0.15 ± 0.01 0.20 ± 0.03 0.33 ± 0.02 0.46 ± 0.06	Sylicylic acid
[61]	LMW	75–85	–	0.2 ^g^	0.084	30	12	–	–	90–315 ^L^	+39.8–+41.8	–	–
[143]	110	85–90	0.1	1.0	1.0	10	1	5	163 ^D^	–	+60.4 ± 4.6	0.42	—
[144]	100–300	–	–	–	–	5:2		3.5	170 ^T^	–	–	–	dsRNA
[55]	50–190	75–85	0.2	1.0 + 1.0 Tween 80	0.2	–	–	–	172.3 ± 0.71 ^D^	–	+49.8 ± 0.75	0.42 ± 0.01	—
									186.9 ± 0.9 ^D^ (GE-NPs 1:0.25) 352.2 ± 1.95 ^D^ (GE-NPs 1:0.75)	–	+32.6 ± 0.60 +27.3 ± 0.80	0.46 ± 0.02 0.51 ± 0.06	Garlic extract (GE)
[145]	244 ± 7	86.9 ± 0.44	0.05	2.0	0.075	40	20	–	180 ^S^	50–800 ^S^	–	–	—
[146]	161	90	0.5	0.5	0.5	3	1	~5	180 ^D^	two peaks	+45.6	0.31	—
[147]	–	80	0.1	1.0	1.0	1:1		–	192.2 ± 2.5 ^D^	150–390 ^D^	+45.33	0.6	Saponin Cu^2+^
[148]	27	75–85	0.2	0.6	0.1	10	6	4.5	195 ± 1 ^D^	–	–	–	Gibberellic acid
[58]	LMW 35 cps ^c^	91.5	0.05	0.01 0.02 0.05 0.08	0.05	10	2.5–3.5	4.7–4.8	196 ^D^ 138 ^D^ 138 ^D^ 138 ^D^	–	–	0.20 0.03 0.08 0.12	—
				0.02					–	$150–160_{16-25\;{}^{\circ}C}^{D}$ ^h^ $135–150_{10-15\;{}^{\circ}C}^{D}$ ^h^ $130–140_{2-4\;{}^{\circ}C}^{D}$ ^h^	+29–+39	>0.2 0.1–0.15 <0.05	
[149]	70 375	75–85	0.2–0.4	0.02	0.01	3	1	–	197 ^D^ 600 ^D^	–	+42–+54 +50–+52	–	—
[108]	LMW	>75	1.0	1.0	0.4	10	10	–	200 ^D^	–	+20–+30	<0.2	Essential oils
[150]	–	–	0.25 0.5 0.75 1.0	1.0	0.1	2:1		–	238 ^D^ 575 ^D^ 706 ^D^ 1315 ^D^	89–513 ^D^ 224–1230 ^D^ 257–1549 ^D^ 468–2952 ^D^	–	–	—
[151]	22	97	0.3	1.0	0.075	30	20	4.6–4.8	259.4 ± 4.7 ^D^	80–200 ^S^	+41 ± 3	0.28 ± 0.02	—
[152]	–	–	0.3	0.5	0.1	7.5:1		–	275 ^D^	15–30 ^T^	+36.2	–	Ag
[153]	38 ^i^	82	0.4	0.5	1.0	30	1	–	297 ^D^ <500 ^S^	–	–	–	—
[99]	LMW	–	0.1	1.0	1.0	3:1		5.5	310 ^D^	150–680 ^D^ 5–40 ^S^	−20–+30	–	—
[110]	50–190	80	0.4	0.5	0.2	–	–	–	370 ^D^	–	+34.1	0.1	Salicylic acid
[154]	186 ± 16	84.71 ± 0.16	1.0	0.25	0.1	8	4	–	–	418–531 ^D^	–	–	FITC-labeled
[109]	50–190	>85	–	1.0	–	–	–	–	–	480–520 ^D^	+22.7–+27.6	0.18–0.24	Zn, salicylic acid
[155]	–	–	0.2	1.0	0.25	100	20	–	500 ^S^ 254 ^D^	–	+103	–	—
[40]	350	>75	0.2	pH 3.5 ^f^	0.05	2:1 3:1 4:1 5:1 6:1 7:1 8:1		–	531 ± 15 ^D^ 396 ± 10 ^D^ 342 ± 8 ^D^ 458 ± 4 ^D^ 498 ± 7 ^D^ 531 ± 12 ^D^ 615 ± 9 ^D^	–	–	0.10 ^BU^ 0.07 ^NU^ 0.05 ^NU^ 0.08 ^BU^ 0.09 ^BU^ 0.09 ^BU^ 0.08 ^BU^	—
[101]	–	90	4.0	1.0	1.0	50	25	–	534 ± 6 ^D^	–	−3.2	0.54	—
[100]	–	–	0.2–0.3	0.1	0.1–0.2	100	100	–	–	674–885 ^D^	−29–−37	0.77–0.89	*Aloe Vera*
[72]	161 300 810	90 75–85 75	0.5	0.5	0.5	3	1	~5	930 ± 150 ^D^	–	–	–	—

Note “–” No data available. “LMW and HMW” Low- and high-molecular-weight Chl. “NU and BU” Narrow unimodal and broad unimodal PDI. ^a^ In some cases, the review’s authors have recalculated values from percentages and molar concentrations for consistency. ^b^ The superscript indicates the method for estimating ChNPs sizes: X—XRPD, T—TEM, D—DLS, A—AFM, S—SEM, L—LD. ^c^ Viscosity of the standard solution. ^d^ Ascorbic acid was used. ^e^ Citric acid was used. ^f^ Only pH is indicated. ^g^ Acetate buffer with pH 5 was used. ^h^ The temperature of the magnetic stirrer which the preheated Chl solution was placed on is indicated. ^i^ Oleyl chitosan was used.

## References

[1] Litter M.I., Ahmad A. (2023). Industrial Applications of Nanoparticles.

[2] Malik S., Muhammad K., Waheed Y. (2023). Nanotechnology: A revolution in modern industry. Molecules.

[3] Haleem A., Javaid M., Singh R.P., Rab S., Suman R. (2023). Applications of nanotechnology in medical field: A brief review. Glob. Health J..

[4] Khan Y., Sadia H., Ali Shah S.Z., Khan M.N., Shah A.A., Ullah N., Ullah M.F., Bibi H., Bafakeeh O.T., Khedher N.B. (2022). Classification, Synthetic, and Characterization Approaches to Nanoparticles, and Their Applications in Various Fields of Nanotechnology: A Review. Catalysts.

[5] Song Y., Zhao G., Zhang S., Xie C., Li X. (2023). A Light-Thin Chitosan Nanofiber Separator for High-Performance Lithium-Ion Batteries. Polymers.

[6] Wang J., Maier S.A., Tittl A. (2022). Trends in nanophotonics-enabled optofluidic biosensors. Adv. Opt. Mater..

[7] Amoozadeh M., Hariri A., Zarepour A., Khosravi A., Iravani S., Zarrabi A. (2025). Biophotonic (nano) structures: From fundamentals to emerging applications. RSC Adv..

[8] Park T., Leem J.W., Kim Y.L., Lee C.H. (2025). Photonic Nanomaterials for Wearable Health Solutions. Adv. Mater..

[9] Rahman B.A., Viphavakit C., Chitaree R., Ghosh S., Pathak A.K., Verma S., Sakda N. (2022). Optical fiber, nanomaterial, and thz-metasurface-mediated nano-biosensors: A Review. Biosensors.

[10] Kumar S., Wang Z., Zhang W., Liu X., Li M., Li G., Singh R. (2023). Optically active nanomaterials and its biosensing applications—A review. Biosensors.

[11] Altug H., Oh S.H., Maier S.A., Homola J. (2022). Advances and applications of nanophotonic biosensors. Nat. Nanotechnol..

[12] Annu M.M., Tripathi S., Shin D.K. (2024). Biopolymeric Nanocomposites for Wastewater Remediation: An Overview on Recent Progress and Challenges. Polymers.

[13] Zambrano-Zaragoza M.L., Mercado-Silva E., Gutiérrez-Cortez E., Castaño-Tostado E., Quintanar-Guerrero D. (2011). Optimization of nanocapsules preparation by the emulsion–diffusion method for food applications. LWT-Food Sci. Technol..

[14] Bradley E.L., Castle L., Chaudhry Q. (2011). Applications of nanomaterials in food packaging with a consideration of opportunities for developing countries. Trends Food Sci. Technol..

[15] Lee D.S. (2010). Packaging and the microbial shelf life of food. Food Packaging and Shelf Life.

[16] Zhang L., Webster T.J. (2009). Nanotechnology and nanomaterials: Promises for improved tissue regeneration. Nano Today.

[17] Gao J., Xu B. (2009). Applications of nanomaterials inside cells. Nano Today.

[18] Qureshi A., Singh D.K., Dwivedi S. (2018). Nano-fertilizers: A novel way for enhancing nutrient use efficiency and crop productivity. Int. J. Curr. Microbiol. Appl. Sci..

[19] Fabiyi O.A., Ogundele A.V., Mella H.S. (2025). Polysaccharide polymer-based nanoparticles for nano fertilizer and nano pesticides—A Review. Carbohydr. Polym. Technol. Appl..

[20] Mondéjar-López M., López-Jiménez A.J., Gómez-Gómez L., Ahrazem O., García-Martínez J.C., Niza E. (2024). Field Crop Evaluation of Polymeric Nanoparticles of Garlic Extract–Chitosan as Biostimulant Seed Nano-Priming in Cereals and Transcriptomic Insights. Polymers.

[21] Sharma B., Tiwari S., Kumawat K.C., Cardinale M. (2023). Nano-biofertilizers as bio-emerging strategies for sustainable agriculture development: Potentiality and their limitations. Sci. Total Environ..

[22] Hernández-Téllez C.N., Luque-Alcaraz A.G., Núñez-Mexía S.A., Cortez-Rocha M.O., Lizardi-Mendoza J., Rosas-Burgos E.C., Rosas-Durazo A.D.J., Parra-Vergara N.V., Plascencia-Jatomea M. (2022). Relationship between the Antifungal Activity of Chitosan–Capsaicin Nanoparticles and the Oxidative Stress Response on *Aspergillus parasiticus*. Polymers.

[23] Maluin F.N., Hussein M.Z. (2020). Chitosan-based agronanochemicals as a sustainable alternative in crop protection. Molecules.

[24] Komarova T., Ilina I., Taliansky M., Ershova N. (2023). Nanoplatforms for the delivery of nucleic acids into plant cells. Int. J. Mol. Sci..

[25] Pan X., Guo X., Zhai T., Zhang D., Rao W., Cao F., Guan X. (2023). Nanobiopesticides in sustainable agriculture: Developments, challenges, and perspectives. Environ. Sci. Nano.

[26] Yadav A., Yadav K., Abd-Elsalam K.A. (2023). Nanofertilizers: Types, delivery and advantages in agricultural sustainability. Agrochemicals.

[27] Tarakanov R., Shagdarova B., Lyalina T., Zhuikova Y., Il’ina A., Dzhalilov F., Varlamov V. (2023). Protective Properties of Copper-Loaded Chitosan Nanoparticles against Soybean Pathogens *Pseudomonas savastanoi* pv. *glycinea* and *Curtobacterium flaccumfaciens* pv. *flaccumfaciens*. Polymers.

[28] Oh J.H., Park D.H., Joo J.H., Lee J.S. (2015). Recent advances in chemical functionalization of nanoparticles with biomolecules for analytical applications. Anal. Bioanal. Chem..

[29] Muzzarelli R.A.A. (1977). Chitin.

[30] Chandrasekaran M., Kim K., Chun S. (2020). Antibacterial Activity of Chitosan Nanoparticles: A Review. Processes.

[31] Li J., Zhuang S. (2020). Antibacterial activity of chitosan and its derivatives and their interaction mechanism with bacteria: Current state and perspectives. Eur. Polym. J..

[32] Ma Z., Garrido-Maestu Z.L., Jeong A. (2017). Application, mode of action, and in vivo activity of chitosan and its micro- and nanoparticles as antimicrobial agents: A review. Carbohydr. Polym..

[33] Chang S.H., Lin H.T.V., Wu G.J., Tsai G.J. (2015). PH Effects on solubility, zeta potential, and correlation between antibacterial activity and molecular weight of chitosan. Carbohydr. Polym..

[34] Ohya Y., Shiratani M., Kobayashi H., Ouchi T. (1994). Release Behavior of 5-Fluorouracil from Chitosan-Gel Nanospheres Immobilizing 5-Fluorouracil Coated with Polysaccharides and Their Cell Specific Cytotoxicity. J. Macromol. Sci. Part A.

[35] Erbacher P., Zou S., Bettinger T., Steffan A.M., Remy J.S. (1998). Chitosan-based vector/DNA complexes for gene delivery: Biophysical characteristics and transfection ability. Pharm. Res..

[36] Maitra A., Ghosh P., De T., Sahoo S.K. (1999). Process for the Preparation of Highly Monodispersed Polymeric Hydrophilic Nanoparticles. U.S. Patent.

[37] El-Shabouri M.H. (2002). Positively charged nanoparticles for improving the oral bioavailability of cyclosporin-A. Int. J. Pharm..

[38] Brunel F., Vacron L., David L., Domard A., Delair T. (2008). A novel synthesis of chitosan nanoparticles in reverse emulsion. Langmuir.

[39] Hoang N.H., Le Thanh T., Sangpueak R., Treekoon J., Saengchan C., Thepbandit W., Buensanteai N. (2022). Chitosan nanoparticles-based ionic gelation method: A promising candidate for plant disease management. Polymers.

[40] Koukaras E.N., Papadimitriou S.A., Bikiaris D.N., Froudakis G.E. (2012). Insight on the formation of chitosan nanoparticles through ionotropic gelation with tripolyphosphate. Mol. Pharm..

[41] Poshina D.N., Rakshina A.D., Skorik Y.A. (2025). Hydrophobic Chitosan Derivatives for Gene and Drug Delivery in Cancer Therapies. Polysaccharides.

[42] Skorik Y.A., Kritchenkov A.S., Moskalenko Y.E., Golyshev A.A., Raik S.V., Whaley A.K., Vasina L.V., Sonin D.L. (2017). Synthesis of N-succinyl- and N-glutaryl-chitosan derivatives and their antioxidant, antiplatelet, and anticoagulant activity. Carbohydr. Polym..

[43] Tiyaboonchai W. (2003). Chitosan Nanoparticles: A Promising System for Drug Delivery. Naresuan Univ. J..

[44] Sailaja A.K., Amareshwar P., Chakravarty P. (2010). Chitosan nanoparticles as a drug delivery system. Res. J. Pharm. Biol. Chem. Sci..

[45] Bhatt S., Pathak R., Punetha V.D., Punetha M. (2024). Chitosan nanocomposites as a nano-bio tool in phytopathogen control. Carbohydr. Polym..

[46] Shinde N.A., Kawar P.G., Dalvi S.G. (2024). Chitosan-based nanoconjugates: A promising solution for enhancing crops drought-stress resilience and sustainable yield in the face of climate change. Plant Nano Biol..

[47] García-Carrasco M., Valdez-Baro O., Cabanillas-Bojórquez L.A., Bernal-Millán M.J., Rivera-Salas M.M., Gutiérrez-Grijalva E.P., Heredia J.B. (2023). Potential agricultural uses of micro/nano encapsulated chitosan: A review. Macromol.

[48] Riseh R.S., Vazvani M.G., Kennedy J.F. (2023). The application of chitosan as a carrier for fertilizer: A review. Int. J. Biol. Macromol..

[49] Sangwan S., Sharma P., Wati L., Mehta S. (2023). Effect of chitosan nanoparticles on growth and physiology of crop plants. Engineered Nanomaterials for Sustainable Agricultural Production, Soil Improvement and Stress Management.

[50] Balusamy S.R., Rahimi S., Sukweenadhi J., Sunderraj S., Shanmugam R., Thangavelu L., Perumalsamy H. (2022). Chitosan, chitosan nanoparticles and modified chitosan biomaterials, a potential tool to combat salinity stress in plants. Carbohydr. Polym..

[51] Hidangmayum A., Dwivedi P. (2022). Chitosan based nanoformulation for sustainable agriculture with special reference to abiotic stress: A review. J. Polym. Environ..

[52] Ingle P.U., Shende S.S., Shingote P.R., Mishra S.S., Sarda V., Wasule D.L., Gade A. (2022). Chitosan nanoparticles (ChNPs): A versatile growth promoter in modern agricultural production. Heliyon.

[53] Prajapati D., Pal A., Dimkpa C., Singh U., Devi K.A., Choudhary J.L., Saharan V. (2022). Chitosan nanomaterials: A prelim of next-generation fertilizers; existing and future prospects. Carbohydr. Polym..

[54] Chouhan D., Mandal P. (2021). Applications of chitosan and chitosan based metallic nanoparticles in agrosciences—A review. Int. J. Biol. Macromol..

[55] Mondéjar-López M., Rubio-Moraga A., López-Jimenez A.J., Martínez J.C.G., Ahrazem O., Gómez-Gómez L., Niza E. (2022). Chitosan nanoparticles loaded with garlic essential oil: A new alternative to tebuconazole as seed dressing agent. Carbohydr. Polym..

[56] Sreekumar S., Goycoolea F.M., Moerschbacher B.M., Rivera-Rodriguez G.R. (2018). Parameters influencing the size of chitosan–TPP nano- and microparticles. Sci. Rep..

[57] Abdel-Hafez S.M., Hathout R.M., Sammour O.A. (2014). Towards better modeling of chitosan nanoparticles production: Screening different factors and comparing two experimental designs. Int. J. Biol. Macromol..

[58] Fan W., Yan W., Xu Z., Ni H. (2012). Formation mechanism of monodisperse, low molecular weight chitosan nanoparticles by ionic gelation technique. Coll. Surf. B Biointerfaces.

[59] El-Assal M.I., Dalia S. (2022). Optimization of rivastigmine chitosan nanoparticles for neurodegenerative Alzheimer; in vitro and ex vivo characterizations. Int. J. Pharm. Pharm. Sci..

[60] Huang S.Y., Han T., Zhong L., Ma C., Zhou Y., Han X. (2012). Improvement of Ag(I) adsorption onto chitosan/triethanolamine composite sorbent by an ion-imprinted technology. Appl. Surf. Sci..

[61] Fàbregas A., Miñarro M., García-Montoya E., Pérez-Lozano P., Carrillo C., Sarrate R., Sánchez N., Ticó J.R., Suñé-Negre J.M. (2013). Impact of physical parameters on particle size and reaction yield when using the ionic gelation method to obtain cationic polymeric chitosan–tripolyphosphate nanoparticles. Int. J. Pharm..

[62] Calvo P., Remuñan-López C., Vila-Jato J.L., Alonso M.J. (1997). Chitosan and Chitosan/Ethylene Oxide-Propylene Oxide Block Copolymer Nanoparticles as Novel Carriers for Proteins and Vaccines. Pharm. Res..

[63] Loutfy S.A., Alam El-Din H.M., Elberry M.H., Allam N.G., Hasanin M.T.H., Abdellah A.M. (2016). Synthesis, characterization and cytotoxic evaluation of chitosan nanoparticles: In vitro liver cancer model. Adv. Nat. Sci. Nanosci. Nanotechnol..

[64] Qi L., Xu Z., Jiang X., Hu C., Zou X. (2004). Preparation and antibacterial activity of chitosan nanoparticles. Carbohydr. Res..

[65] Dong Y., Ng W.K., Shen S., Kim S., Tan R.B.H. (2013). Scalable ionic gelation synthesis of chitosan nanoparticles for drug delivery in static mixers. Carbohydr. Polym..

[66] Elshayb O.M., Ghazy H.A., Wissa M.T., Farroh K.Y., Wasonga D.O., Seleiman M.F. (2024). Chitosan-based NPK nanostructure for reducing synthetic NPK fertilizers and improving rice productivity and nutritional indices. Front. Sustain. Food Syst..

[67] Pham T.T., Nguyen T.H., Thi T.V., Nguyen T.-T., Le T.D., Vo D.M.H., Nguyen D.H., Nguyen C.K., Nguyen D.C., Nguyen T.T. (2019). Investigation of Chitosan Nanoparticles Loaded with Protocatechuic Acid (PCA) for the Resistance of *Pyricularia oryzae* Fungus against Rice Blast. Polymers.

[68] Ing B.Y., Zin N.M., Sarwar A., Katas H. (2012). Antifungal Activity of Chitosan Nanoparticles and Correlation with Their Physical Properties. Int. J. Biomater..

[69] Du W.-L., Niu S.-S., Xu Y.-L., Xu Z.-R., Fan C.-L. (2009). Antibacterial activity of chitosan tripolyphosphate nanoparticles loaded with various metal ions. Carbohydr. Polym..

[70] Hu B., Pan C., Sun Y., Hou Z., Ye H., Hu B., Zeng X. (2008). Optimization of Fabrication Parameters To Produce Chitosan-Tripolyphosphate Nanoparticles for Delivery of Tea Catechins. J. Agric. Food Chem..

[71] Chauhan N., Dilbaghi N., Gopal M., Kumar R., Kim K.-H., Kumar S. (2017). Development of Chitosan Nanocapsules for the Controlled Release of Hexaconazole. Int. J. Biol. Macromol..

[72] Kheiri A., Jorf S.A.M., Malihipour A., Saremic H., Nikkhah M. (2017). Synthesis and characterization of chitosan nanoparticles and their effect on Fusarium head blight and oxidative activity in wheat. Int. J. Biol. Macromol..

[73] Mohammadi A., Hashemi M., Masoud Hosseini S. (2016). Effect of chitosan molecular weight as micro and nanoparticles on antibacterial activity against some soft rot pathogenic bacteria. LWT Food Sci. Technol..

[74] Jonassen H., Kjøniksen A.-L., Hiorth M. (2012). Stability of Chitosan Nanoparticles Cross-Linked with Tripolyphosphate. Biomacromolecules.

[75] Divya K., Vijayan S., Nair S.J., Jisha M.S. (2019). Optimization of chitosan nanoparticle synthesis and its potential application as germination elicitor of *Oryza sativa* L. Int. J. Biol. Macromol..

[76] Handani W.R., Sediawan W.B., Tawfiequrrahman A., Kusumastuti W. (2017). The effect of temperature and chitosan concentration during storage on the growth of chitosan nanoparticle produced by ionic gelation method. AIP Conf. Proc..

[77] Kleine-Brueggeney H., Zorzi G.K., Fecker T., El-Gueddari N.E., Moerschbacher B.M., Goycoolea F.M. (2015). A rational approach towards the design of chitosan-based nanoparticles obtained by ionotropic gelation. Coll. Surf. B Biointerfaces.

[78] Morris G.A., Castile J., Smith A., Adams G.G., Harding S.E. (2011). The effect of prolonged storage at different temperatures on the particle size distribution of tripolyphosphate (TPP)–chitosan nanoparticles. Carbohydr. Polym..

[79] Huang Y., Cai Y., Lapitsky Y. (2015). Factors Affecting the Stability of Chitosan/Tripolyphosphate Micro- and Nanogels: Resolving the Opposing Findings. J. Mater. Chem. B.

[80] Yang H.-C., Hon M.-H. (2010). The Effect of the Degree of Deacetylation of Chitosan Nanoparticles and its Characterization and Encapsulation Efficiency on Drug Delivery. Polym. Plast. Technol. Eng..

[81] Goycoolea F.M., Brunel F., Gueddari N.E.E., Coggiola A., Lollo G., Moerschbacher B.M., Alonso M.J. (2016). Physical properties and stability of soft gelled chitosan-based nanoparticles. Macromol. Biosci..

[82] Antoniou J., Liu F., Majeed H., Qi J., Yokoyama W., Zhong F. (2015). Physicochemical and morphological properties of size-controlled chitosan–tripolyphosphate nanoparticles. Coll. Surf. A Physicochem. Eng. Asp..

[83] Bulmer C., Margaritis A., Xenocostas A. (2012). Production and Characterization of Novel Chitosan Nanoparticles for Controlled Release of rHu-Erythropoietin. Biochem. Eng. J..

[84] Ellison H., Martell A.E. (1964). Chelating tendencies of tripolyphosphate ions. J. Inorg. Nucl. Chem..

[85] Edwards O.W., Farr T.D., Dunn R.L., Hatfield J.D. (1973). Dissociation constants of pyro-and tripolyphosphoric acids at 25°. J. Chem. Eng. Data.

[86] Bhumkar D.R., Pokharkar V.B. (2006). Studies on effect of pH on cross-linking of chitosan with sodium tripolyphosphate: A technical note. AAPS PharmSciTech.

[87] Hashad R.A., Ishak R.A., Fahmy S., Mansour S., Geneidi A.S. (2016). Chitosan-tripolyphosphate nanoparticles: Optimization of formulation parameters for improving process yield at a novel pH using artificial neural networks. Int. J. Biol. Macromol..

[88] Mazancová P., Némethová V., Treľová D., Kleščíková L., Lacík I., Rázga F. (2018). Dissociation of chitosan/tripolyphosphate complexes into separate components upon pH elevation. Carbohydr. Polym..

[89] Gan Q., Wang T., Cochrane C., McCarron P. (2005). Modulation of surface charge, particle size and morphological properties of chitosan–TPP nanoparticles intended for gene delivery. Colloids Surf. B Biointerfaces.

[90] López-León T., Carvalho E.L.S., Seijo B., Ortega-Vinuesa J.L., Bastos-González D. (2005). Physicochemical characterization of chitosan nanoparticles: Electrokinetic and stability behavior. J. Colloid Interface Sci..

[91] Raj P.M., Raj R., Kaul A., Mishra A.K., Ram A. (2018). / Biodistribution and targeting potential assessment of mucoadhesive chitosan nanoparticles designed for ulcerative colitisviascintigraphy. RSC Adv..

[92] Cai Y., Lapitsky Y. (2020). Biomolecular uptake effects on chitosan/tripolyphosphate micro- and nanoparticle stability. Colloids Surf. B Biointerfaces.

[93] He P., Davis S.S., Illum L. (1999). Chitosan microspheres prepared by spray drying. Int. J. Pharm..

[94] Bodnar M., Hartmann J.F., Borbely J. (2005). Preparation and Characterization of Chitosan-Based Nanoparticles. Biomacromolecules.

[95] Gokce Y., Cengiz B., Yildiz N., Calimli A., Aktas Z. (2014). Ultrasonication of chitosan nanoparticle suspension: Influence on particle size. Colloids Surf. A Physicochem. Eng. Asp..

[96] Oudih S.B., Tahtat D., Khodja A.N., Mahlous M., Hammache Y., Guittoum A.-E., Gana S.K. (2023). Chitosan nanoparticles with controlled size and zeta potential. Polym. Eng. Sci..

[97] Al-Nemrawi N.K., Alsharif S.S.M., Dave R.H. (2018). Preparation of chitosan-TPP nanoparticles: The influence of chitosan polymeric properties and formulation variables. Int. J. Appl. Pharm..

[98] de Pinho Neves A.L., Milioli C.C., Müller L., Riella H.G., Kuhnen N.C., Stulzer H.K. (2014). Factorial design as tool in chitosan nanoparticles development by ionic gelation technique. Colloids Surf. A Physicochem. Eng. Asp..

[99] Anju A.B., Gopal K.S., Panchami P.S., Vijayaraghavan R. (2025). A simple and user-friendly protocol for chitosan nanoparticle synthesis. Discov. Nano.

[100] Efrilia E., Sunarni T., Kuncahyo I. (2025). Optimization of chitosan and sodium tripolyphosphate as a carrier system for nanoparticles of ethanol extract of aloe vera (*Aloe vera* L.) as an antioxidant. J. Health Manag. Pharm. Explor..

[101] Badawy M.E.I., Rabea E.I., Eid A.R., Badr M.M., Marei G.I.K. (2021). Structure and antimicrobial comparison between N-(benzyl) chitosan derivatives and N-(benzyl) chitosan tripolyphosphate nanoparticles against bacteria, fungi, and yeast. Int. J. Biol. Macromol..

[102] Aleanizy F.S., Alqahtani F.Y., Shazly G., Alfaraj R., Alsarra I., Alshamsan A., Abdulhady H.G. (2018). Measurement and evaluation of the effects of pH gradients on the antimicrobial and antivirulence activities of chitosan nanoparticles in *Pseudomonas aeruginosa*. Saudi Pharm. J..

[103] Manikandan A., Sathiyabama M. (2016). Preparation of chitosan nanoparticles and its effect on detached rice leaves infected with *Pyricularia grisea*. Int. J. Biol. Macromol..

[104] Sathiyabama M., Muthukumar S. (2020). Chitosan guar nanoparticle preparation and its in vitro antimicrobial activity towards phytopathogens of rice. Int. J. Biol. Macromol..

[105] Gonçalves M.M., Maluf D.F., Pontarolo R., Saul C.K., Almouazen E., Chevalier Y. (2023). Negatively charged chitosan nanoparticles prepared by ionotropic gelation for encapsulation of positively charged proteins. Int. J. Pharm..

[106] Rázga F., Vnukova D., Nemethova V., Mazancova P., Lacik I. (2016). Preparation of chitosan-TPP sub-micron particles: Critical evaluation and derived recommendations. Carbohydr. Polym..

[107] Desai K.G. (2016). Chitosan Nanoparticles Prepared by Ionotropic Gelation: An Overview of Recent Advances. Crit. Rev. Ther. Drug Carr. Syst..

[108] Machado S., Ganilho C., Andreani T., Sousa R.M.O.F., Ribeiro A., Pereira R. (2026). Chitosan/Tripolyphosphate Nanoparticles Encapsulating Essential Oils as a New Class of Biopesticides: Structural Properties and Ecotoxicity Evaluation. Environ. Toxicol..

[109] Mohan N., Pal A., Saharan V., Kumar A., Vashishth R., Prince S.E. (2024). Development, characterization, and evaluation of Zn-SA-chitosan bionanoconjugates on wheat seed, experiencing chilling stress during germination. Heliyon.

[110] Kumaraswamy R.V., Kumari S., Choudhary R.C., Sharma S.S., Pal A., Raliya R., Biswas P., Saharan V. (2018). Salicylic acid functionalized chitosan nanoparticle: A sustainable biostimulant for plant. Int. J. Biol. Macromol..

[111] Oh J.-W., Chun S.C., Chandrasekaran M. (2019). Preparation and In Vitro Characterization of Chitosan Nanoparticles and Their Broad-Spectrum Antifungal Action Compared to Antibacterial Activities against Phytopathogens of Tomato. Agronomy.

[112] Ke C.-L., Deng F.-S., Chuang C.-Y., Lin C.-H. (2021). Antimicrobial Actions and Applications of Chitosan. Polymers.

[113] Liang W., Yu A., Wang G., Zheng F., Hu P., Jia J., Xu H. (2018). A novel water-based chitosan-La pesticide nanocarrier enhancing defense responses in rice (*Oryza sativa* L.) growth. Carbohydr. Polym..

[114] Xing K., Zhu X., Peng X., Qin S. (2014). Chitosan antimicrobial and eliciting properties for pest control in agriculture: A review. Agron. Sustain. Dev..

[115] Arpa M.D., Erim Ü.C., Kesmen Salik E.E., Kaleli S.N.B., Erol I. (2025). Effect of Organic Acid Selection on the Physicochemical Properties, Bioadhesion, and Stability of Chitosan Hydrogels. Gels.

[116] Malinkina O.N., Shmakov S.L., Shipovskaya A.B. (2024). Structure, the energy, sorption and biological properties of chiral salts of chitosan with L- and D-ascorbic acid. Int. J. Biol. Macromol..

[117] Gegel N.O., Zhuravleva Y.Y., Shipovskaya A.B., Malinkina O.N., Zudina I.V. (2018). Influence of chitosan ascorbate chirality on the gelation kinetics and properties of silicon-chitosan-containing glycerohydrogels. Polymers.

[118] Singh J., Dutta P.K. (2009). Preparation, circular dichroism induced helical conformation and optical property of chitosan acid salt complexes for biomedical applications. Int. J. Biol. Macromol..

[119] Pirniyazov K.K., Asrakulova D.I., Rashidova S.S. (2024). Synthesis and antimicrobial properties of chitosan nanoascorbate *Bombyx mori*. Mosc. Univ. Chem. Bull..

[120] Hassan E.O., Shoala T., Attia A.M.F., Badr O.A.M., Mahmoud S.Y.M., Farrag E.S.H., EL-Fiki I.A.I. (2022). Chitosan and Nano-Chitosan for Management of *Harpophora maydis*: Approaches for Investigating Antifungal Activity, Pathogenicity, Maize-Resistant Lines, and Molecular Diagnosis of Plant Infection. J. Fungi.

[121] Luangtana-Anan M., Nunthanid J., Limmatvapirat S. (2019). Potential of different salt forming agents on the formation of chitosan nanoparticles as carriers for protein drug delivery systems. J. Pharm. Investig..

[122] Pilon L., Spricigo P.C., Miranda M., de Moura M.R., Assis O.B.G., Mattoso L.H.C., Ferreira M.D. (2015). Chitosan nanoparticle coatings reduce microbial growth on fresh-cut apples while not affecting quality attributes. Int. J. Food Sci. Technol..

[123] Lugovitskaya T.N., Shipovskaya A.B., Shmakov S.L., Shipenok X.M. (2022). Formation, structure, properties of chitosan aspartate and metastable state of its solutions for obtaining nanoparticles. Carbohydr. Polym..

[124] Lugovitskaya T.N., Shipovskaya A.B., Shipenok X.M. (2021). Kinetic instability of a chitosan—Aspartic acid—Water system as a method for obtaining nano- and microparticle. Chim. Techno Acta.

[125] Shipovskaya A.B., Ushakova O.S., Volchkov S.S., Shipenok X.M., Shmakov S.L., Gegel N.O., Burov A.M. (2024). Chiral nanostructured glycerohydrogel sol–gel plates of chitosan L- and D-aspartate: Supramolecular ordering and optical properties. Gels.

[126] Shipenok K.M., Lugovitskaya T.N., Shipovskaya A.B. (2024). Structure Formation during the Synthesis of Chitosan L- and D-Asparaginate Nanoparticles. Russ. J. Phys. Chem. A.

[127] Shipovskaya A.B., Lugovitskaya T.N., Zudina I.V. (2023). Biocidal Activity of Chitosan Aspartate Nanoparticles. Microbiology.

[128] Shipovskaya A., Shipenok X., Lugovitskaya T., Babicheva T. (2023). Self-assembling nano- and microparticles of chitosan L-and D-aspartate: Preparation, structure, and biological activity. Mater. Proc..

[129] Tkachenko O.V., Pozdnyakova N.N., Kostina E.E., Shcherbakova E.V., Shipenok X.M., Shipovskaya A.B. (2025). Antifungal Activity of Chitosan Asparaginate Shell Nanoparticles. Microbiology.

[130] Tkachenko O.V., Kargopolova K.Y., Denisova A.Y., Shipenok K.M., Shipovskaya A.B. Biopreparation for Stimulating Growth and Development of Plants and Inhibiting Phytopathogens. RU2841251C1, 4 June 2025. https://patents.google.com/patent/RU2841251C1.

[131] Ismail A.M., Mosa M.A., El-Ganainy S.M. (2023). Chitosan-Decorated Copper Oxide Nanocomposite: Investigation of Its Antifungal Activity against Tomato Gray Mold Caused by *Botrytis cinerea*. Polymers.

[132] Paulraj M.G., Ignacimuthu S., Gandhi M.R., Shajahan A., Ganesan P., Packiam S.M., Al-Dhabi N.A. (2017). Comparative studies of Tripolyphosphate and Glutaraldehyde cross-linked chitosan botanical pesticide nanoparticles and their agricultural applications. Int. J. Biol. Macromol..

[133] Oliveira H.C., Gomes B.C.R., Pelegrino M.T., Seabra A.B. (2016). Nitric oxide-releasing chitosan nanoparticles alleviate the effects of salt stress in maize plants. Nitric Oxide.

[134] Shirkhani Z., Rad A.C., Mohsenzadeh F. (2021). Improving Cd-phytoremediation ability of *Datura stramonium* L. by Chitosan and Chitosan nanoparticles. Biologia.

[135] El-Mohamedy R.S.R., El-Aziz M.E.A., Kamel S. (2019). Antifungal activity of chitosan nanoparticles against some plant pathogenic fungi in vitro. Agric. Eng. Int. CIGR J..

[136] El-Aziz M.E.A., Morsi S., Salama D.M., Abdel-Aziz M.S., Elwahed M.S.A., Shaaban E.A., Youssef A.M. (2019). Preparation and characterization of chitosan/polyacrylic acid/copper nanocomposites and their impact on onion production. Int. J. Biol. Macromol..

[137] Elkeiy M.M., Khamis A.A., El-Gamal M.M., Abo Gazia M.M., Zalat Z.A., El-Magd M.A. (2020). Chitosan nanoparticles from *Artemia salina* inhibit progression of hepatocellular carcinoma in vitro and in vivo. Environ. Sci. Pollut. Res..

[138] Chandra S., Chakraborty N., Dasgupta A., Sarkar J., Panda K., Acharya K. (2015). Chitosan nanoparticles: A positive modulator of innate immune responses in plants. Sci. Rep..

[139] Kain D., Kumar S. (2020). Synthesis and characterization of chitosan nanoparticles of *Achillea millefolium* L. and their activities. F1000Research.

[140] Ramírez-Rodríguez S.C., Rangel P.P., La Fuente M.C.D., González-Morales S., Ortega-Ortiz H. (2024). Chitosan Nanoparticles as Biostimulant in Lettuce (*Lactuca sativa* L.) Plants. Phyton.

[141] Lustriane C., Dwivany F.M., Suendo V., Reza M. (2018). Effect of chitosan and chitosan nanoparticles on post harvest quality of banana fruits. J. Plant Biotechnol..

[142] Valderrama A.N., Jacinto C.H., Lay J., Flores Y.E., Zavaleta D.C., Delfín A.R. (2020). Factorial design for preparing chitosan nanoparticles and its use for loading and controlled release of indole-3-acetic acid with effect on hydroponic lettuce crops. Biocatal. Agric. Biotechnol..

[143] Asgari-Targhia G., Iranbakhsha A., Ardebili Z.O. (2018). Potential benefits and phytotoxicity of bulk and nano-chitosan on the growth, morphogenesis, physiology, and micropropagation of *Capsicum annuum*. Plant Physiol. Biochem..

[144] Scarpin D., Nerva L., Chitarra W., Moffa L., D’Este F., Vuerich M., Filippi A., Braidot E., Petrussa E. (2023). Characterisation and functionalisation of chitosan nanoparticles as carriers for double-stranded RNA (dsRNA) molecules towards sustainable crop protection. Biosci. Rep..

[145] Abdel-Aliem H.A., Gibriel A.Y., Rasmy N.M.H., Sahab A.F., El-Nekeety A.A., Abdel-Wahhab M.A. (2019). Antifungal efficacy of chitosan nanoparticles against phytopathogenic fungi and inhibition of zearalenone production by *Fusarium graminearum*. Comun. Sci..

[146] Kheiri A., Moosawijorf S.A., Mallihipour A., Saremi H., Nikkhah M. (2016). Application of chitosan and chitosan nanoparticles for the control of Fusarium head blight of wheat (*Fusarium graminearum*) in vitro and greenhouse. Int. J. Biol. Macromol..

[147] Saharan V., Mehrotra A., Khatik R., Rawal P., Sharma S.S., Pal A. (2013). Synthesis of chitosan based nanoparticles and their in vitro evaluation against phytopathogenic fungi. Int. J. Biol. Macromol..

[148] Pereira A.E.S., Silva P.M., Oliveira J.L., Oliveira H.C., Fraceto L.F. (2017). Chitosan nanoparticles as carrier systems for the plant growth hormone gibberellic acid. Colloids Surf. B Biointerfaces.

[149] Sarwar A., Katas H., Zin N.M. (2014). Antibacterial effects of chitosan–tripolyphosphate nanoparticles: Impact of particle size molecular weight. J. Nanopart. Res..

[150] Bangun H., Tandiono S., Arianto A. (2018). Preparation and evaluation of chitosan-tripolyphosphate nanoparticles suspension as an antibacterial agent. J. Appl. Pharm. Sci..

[151] Li R., He J., Xie H., Wang W., Kumar B.S., Sun Y., Hu J., Yin H. (2019). Effects of chitosan nanoparticles on seed germination and seedling growth of wheat (*Triticum aestivum* L.). Int. J. Biol. Macromol..

[152] Dananjaya S.H.S., Erandani W.K.C.U., Kim C.-H., Nikapitiya C., Lee J., De Zoysa M. (2017). Comparative study on antifungal activities of chitosan nanoparticles and chitosan silver nano composites against *Fusarium oxysporum* species complex. Int. J. Biol. Macromol..

[153] Xing K., Shen X., Zhu X., Ju X., Miao X., Tian J., Feng Z., Peng X., Jiang J., Qin S. (2016). Synthesis and in vitro antifungal efficacy of oleoyl-chitosan nanoparticles against plant pathogenic fungi. Int. J. Biol. Macromol..

[154] Ma Z., Lim L.-Y. (2003). Uptake of Chitosan and Associated Insulin in Caco-2 Cell Monolayers: A Comparison Between Chitosan Molecules and Chitosan Nanoparticles. Pharm. Res..

[155] Sen S.K., Chouhan D., Das D., Ghosh R., Mandal P. (2020). Improvisation of salinity stress response in mung bean through solid matrix priming with normal and nano-sized chitosan. Int. J. Biol. Macromol..

