# Supplementary material for: A Critical Review of Research on the Production and Properties of Chitosan Nanoparticles, Promising for Agrobiotechnology, Obtained Through Ionic Gelation with Sodium Tripolyphosphate
Source: Polymers (Basel). 2026 Jul 6;18(13):1668. doi: 10.3390/polym18131668 (PMC13364282; doi:10.3390/polym18131668)
Supplement: Supplementary file 1 [file polymers-18-01668-s001.zip › polymers-4382834-supplementary.pdf]

Table S1. Recent review articles on chitosan nanobiochemicals

Year	First Author	Title	Journal
2024	Bhatt S.	Chitosan nanocomposites as a nano-bio tool in phytopathogen control	Carbohydrate Polymers
2024	Chowardhara B.	An assessment of nanotechnology-based interventions for cleaning up toxic heavy metal/metalloid-contaminated agroecosystems: Potentials and issues	Chemosphere
2024	Das A.	Chapter 8. Chitosan Biopolymer Nanocomposites for Agriculture Applications	In: Biopolymeric Nanoparticles for Agricultural Applications
2024	El-Araby A.	Chitosan, chitosan derivatives, and chitosan-based nanocomposites: eco-friendly materials for advanced applications (a review)	Front. Chem.
2024	Rameez M.	Bionanocomposites: A new approach for fungal disease management	Biocatalysis and Agricultural Biotechnology
2024	Rivera-Solırs L.L.	Chapter 8. Biostimulation of plants with nanocomposites: a new perspective to improve crop production	In: Nanocomposites for Environmental, Energy, and Agricultural Applications
2024	Rojas-Pirela M.	Effects of chitosan on plant growth under stress conditions: similarities with plant growth promoting bacteria	Front. Plant Sci.
2024	Shinde N.A.	Chitosan-based nanoconjugates: A promising solution for enhancing crops drought-stress resilience and sustainable yield in the face of climate change	Plant Nano Biology
2024	Soni S.K.	Enhancing Crop Productivity with Sustainable Nano-Fertilizers and Nano-Biofertilizers	Journal of Soil Science and Plant Nutrition
2024	Sreelakshmi P.	Chitosan and Its Derivatives for Agriculture Applications: A Review	International Journal of Plant & Soil Science
2024	Suwanchaikasem P.	The Impacts of Chitosan on Plant Root Systems and Its Potential to be Used for Controlling Fungal Diseases in Agriculture	Journal of Plant Growth Regulation
2024	Virk V.	Amelioration in nanobiosensors for the control of plant diseases: current status and future challenges	Front. Nanotechnol.
2024	Wang X.	Emerging Nanochitosan for Sustainable Agriculture	Int. J. Mol. Sci.
2023	Garcна-Carrasco M.	Potential Agricultural Uses of Micro/Nano Encapsulated Chitosan: A Review	Macromol.
2023	Haris M.	Nanotechnology – A new frontier of nano-farming in agricultural and food production and its development	Science of the Total Environment
2023	Komarova T.	Nanoplatforms for the Delivery of Nucleic Acids into Plant Cells	Int. J. Mol. Sci.
2023	Nirmala M.J.	Chapter. Chitosan-Based Nanofertilizer: Types, Formulations, and Plant Promotion Mechanism	In: Nanofertilizers for Sustainable Agroecosystems
2023	Omar R.A.	Chapter. Nanostructure-Based Smart Fertilizers and Their Interaction with Plants	
2023	Pan X.	Nanobiopesticides in sustainable agriculture: developments, challenges, and perspectives	Environ. Sci.: Nano
2023	Poznanski P.	Chitosan and Chitosan Nanoparticles: Parameters Enhancing Antifungal Activity	Molecules
2023	Riseh R.	The application of chitosan as a carrier for fertilizer: A review	International Journal of Biological Macromolecules
2023	Román-Doval R.	Chitosan: Properties and Its Application in Agriculture in Context of Molecular Weight	Polymers

Year	First Author	Title	Journal
2023	Sangwan S.	Effect of chitosan nanoparticles on growth and physiology of crop plants.	In: Engineered Nanomaterials for Suatainable Agricultural Production, Soil Improvement and Stress Management
2023	Sharma B.	Nano-biofertilizers as bio-emerging strategies for sustainable agriculture development: Potentiality and their limitations	Science of the Total Environment
2023	Sun W.	Developing Sustainable Agriculture Systems in Medicinal and Aromatic Plant Production by Using Chitosan and Chitin-Based Biostimulants	Plants
2023	Yadav A.	Nanofertilizers: Types, Delivery and Advantages in Agricultural Sustainability	Agrochemicals
2022	Ashraf U.	Chapter 7. Impact of nano chitosan-NPK fertilizer on field crops	In: Role of Chitosan and Chitosan-Based Nanomaterials in Plant Sciences
2022	González-García Y.	Chapter 12. Impact of chitosan and chitosan based nanoparticles on plants growth and development	
2022	Balusamy S.R.	Chitosan, chitosan nanoparticles and modi 香 ed chitosan biomaterials, a potential tool to combat salinity stress in plants	Carbohydrate Polymers
2022	Hidangmayum A.	Chitosan Based Nanoformulation for Sustainable Agriculture with Special Reference to Abiotic Stress: A Review	Journal of Polymers and the Environment
2022	Hoang N.H.	Chitosan Nanoparticles-Based Ionic Gelation Method: A Promising Candidate for Plant Disease Management	Polymers
2022	Ilmudeen S.	Advances of Nanofertilizers in Modern Agriculture; A review	J. Res. Technol. Eng.
2022	Ingle P.U.	Chitosan nanoparticles (ChNPs): A versatile growth promoter in modern agricultural production	Heliyon
2022	Ji H.	Meta-analysis of chitosan-mediated effects on plant defense against oxidative stress	Science of the Total Environment
2022	Karamchandani B.M.	Chitosan and its derivatives: Promising biomaterial in averting fungal diseases of sugarcane and other crops	Journal of Basic Microbiology
2022	Mishra K.K.	Chapter 20. Chitosan and chitosan-based nanoparticles for eco-friendly management of plant diseases and insect pests: a concentric overview	Book: Role of Chitosan and Chitosan-Based Nanomaterials in Plant Sciences
2022	Prajapati D.	Chitosan nanomaterials: A prelim of next-generation fertilizers; existing and future prospects. Review	Carbohydrate Polymers
2022	Sharma S.	Recent developments in smart nano-agrochemicals: A promise for revolutionizing present-day agriculture	Materials Today: Proceedings
2022	Stasińska-Jakubas M.	Protective, Biostimulating, and Eliciting Effects of Chitosan and Its Derivatives on Crop Plants	Molecules
2022	Zhang M.	Application of Chitosan and Its Derivative Polymers in Clinical Medicine and Agriculture	Polymers
2021	Azmana M.	A review on chitosan and chitosan-based bionanocomposites: Promising material for combatting global issues and its applications	International Journal of Biological Macromolecule
2021	Chouhan D.	Applications of chitosan and chitosan based metallic nanoparticles in agrosciences — A review	International Journal of Biological Macromolecules
2021	Faqir Y.	Chitosan in modern agriculture production /	Plant, Soil and Environment
2021	Faqir Y.	Application of Chitosan in Plant Growth	Adv. Crop. Sci. Tech.

Year	First Author	Title	Journal
2021	Fellet G.	Tools for Nano-Enabled Agriculture: Fertilizers Based on Calcium Phosphate, Silicon, and Chitosan Nanostructures	Agronomy
2021	Hamrayev H.	Green Route for the Fabrication of ZnO Nanoparticles and Potential Functionalization with Chitosan Using Cross-linkers: A Review	Journal of Research in Nanoscience and Nanotechnology
2021	Kocięcka J.	The Potential of Using Chitosan on Cereal Crops in the Face of Climate Change	Plants
2021	Kumar A.	Smart nanomaterial and nanocomposite with advanced agrochemical activities	Nanoscale Res. Lett.
2021	Shahrajabian M.H.	Sustainable Agriculture Systems in Vegetable Production Using Chitin and Chitosan as Plant Biostimulants	Biomolecules
2021	Silva A.O.	Chitosan as a matrix of nanocomposites: A review on nanostructures, processes, properties, and applications	Carbohydrate Polymers
2021	Singh S.	Nanotechnology for Sustainable Agriculture: An Emerging Perspective	Journal of Nanoscience and Nanotechnology
2021	Yu J.	Current trends and challenges in the synthesis and applications of chitosan-based nanocomposites for plants: A review	Carbohydrate Polymers
2020	Bandara S.	Agricultural and Biomedical Applications of Chitosan-Based Nanomaterials	Nanomaterials
2020	Chandrasekaran M.	Antibacterial Activity of Chitosan Nanoparticles: A Review	Processes
2020	Maluin F.N.	Chitosan-Based Agronanochemicals as a Sustainable Alternative in Crop Protection	Molecules
2020	Mujtaba M.	Chitosan-based delivery systems for plants: A brief overview of recent advances and future directions	International Journal of Biological Macromolecules
2020	Oliveira A.M.	Antifungal and filmogenic properties of micro- and nanostructures of chitosan and its derivatives	Nanomycotoxicology
2020	Rani T.S.	Chapter 17. Chitosan conjugates, microspheres, and nanoparticles with potential agrochemical activity	Book: Agrochemicals Detection, Treatment and Remediation
2020	Somdutt H.J.	Controlled release action of chitosan nanoparticles to improve nutrient use efficiency	International Journal of Research in Agronomy
2020	Xin X.	Nano-enabled agriculture: from nanoparticles to smart nanodelivery systems	Environ. Chem.
2020	Mujtaba M.	Chitosan-based delivery systems for plants: A brief overview of recent advances and future directions	International journal of biological macromolecules
2019	Sanzari I.	Nanotechnology in Plant Science: To Make a Long Story Short	Front. Bioeng. Biotechnol.
2018	Divya K.	Chitosan nanoparticles preparation and applications	Environ Chem Lett
2018	Kumaraswamy R.V.	Engineered chitosan-based nanomaterials: Bioactivities, mechanisms and perspectives in plant protection and growth	International Journal of Biological Macromolecules
2018	Worrall E.A.	Nanotechnology for Plant Disease Management	Agronomy
2017	Aljebory A.M.	Chitosan Nanoparticles: Review Article	Imperial Journal of Interdisciplinary Research
2017	Ma Z.	Application, Mode of Action, and In Vivo Activity of Chitosan and its Micro- and Nanoparticles as Antimicrobial Agents: A Review	Carbohydrate Polymers
2016	Malerba M.	Chitosan Effects on Plant Systems	Int. J.of Molecular Sciences
2016	Shalaby T.	Chapter 10. Nanoparticles, Soils, Plants and Sustainable Agriculture	In: Nanoscience in Food and Agriculture

Year	First Author	Title	Journal
2015	Kashyap P.L.	Chitosan nanoparticle based delivery systems for sustainable agriculture	Macromolecules
2015	Katiyar D.	Chitosan as a promising natural compound to enhance potential physiological responses in plant: a review	Ind J Plant Physiol.
2014	Arruda S.	Nanoparticles Applied to Plant Science: A Review	Talanta
2014	Mukhopadhyay S.	Nanotechnology in agriculture: prospects and constraints	Nanotechnology, Science and Applications
2013	Shukla S.K.	Chitosan-based nanomaterials: A state-of-the-art review	International Journal of Biological Macromolecules
2012	Cota-Arriola O.	Controlled release matrices and micro/nanoparticles of chitosan with antimicrobial potential: development of new strategies for microbial control in agriculture	J Sci Food Agric
2012	Grenha A.	Chitosan nanoparticles: a survey of preparation methods	Journal of Drug Targeting
2012	Khot L.R.	Applications of nanomaterials in agricultural production and crop protection: A review	Crop Protection
2011	Zhao L.M.	Preparation and application of chitosan nanoparticles and nanofibers	Brazilian Journal of Chemical Engineering

Table S2. Comparison of different ChNP preparation methods

Method	Advantages	Limitations	Characteristics
Polyelectrolyte complex formation method	Environmentally friendly, no toxic byproducts, gentle bio-encapsulation, stimulus-responsive control, scalable and modifiable, enhanced material strength	Poor processability, environmental instability, batch-to-batch inconsistencies	Spontaneous electrostatic attraction between oppositely charged polymers in aqueous solutions
Microemulsion method	Spontaneous formation, excellent thermodynamic stability, enhanced solubilization of both water- and oil-soluble compounds	High requirement for surfactants/co-surfactants, strict temperature and pH sensitivity, restricted solubilizing capacity	Using surfactants to lower surface tension, creating "nanoreactors" that naturally control the size, shape, and distribution of particles while preventing them from clumping together
Emulsification solvent diffusion method	Ability to produce incredibly small, uniform nanoparticles with high encapsulation efficiency while avoiding toxic, harsh processing conditions like high heat or intense mechanical stress	All limitations span from processing complexity to material restrictions, which often impede its widespread industrial application	Diffusion of a partially water-miscible organic solvent from an emulsified droplet into a surrounding aqueous phase, causing the dissolved polymer or lipid to precipitate into solid nanoparticles
Coacervation and reverse micellar method	High efficiency, no toxic solvents, superior protection, controlled release, masking capabilities	Strict environmental sensitivity, limited payload capacity, scalability challenges	Both methods involve self-assembly, but operate under completely opposite principles
Ionic gelation method	Mild aqueous processing, non-toxicity, excellent controlled-release capabilities	Uncontrolled cross-linking leading to wide size variations (polydispersity), poor stability in acidic conditions, and difficulty in scaling up to commercial production	Creating micro- or nanoparticles by cross-linking polyelectrolytes with oppositely charged ions
Hydrophobic chain	Permanent covalent bonding, enhanced anti-fouling,	Compromised bulk properties, synthetic	Chemical modification to attach water-repellent

grafting method	superior separation efficiency, material versatility, tunable properties	bottlenecks, environmental instability	(hydrophobic) molecules or polymer chains to a surface or a main polymer backbone
